# Lipid microdomain modification sustains neuronal viability in models of Alzheimer’s disease

**DOI:** 10.1186/s40478-016-0354-z

**Published:** 2016-09-17

**Authors:** Silke Herzer, Sascha Meldner, Klara Rehder, Hermann-Josef Gröne, Viola Nordström

**Affiliations:** 1 Department of Cellular and Molecular Pathology, German Cancer Research Center, 69120 Heidelberg, Germany; 2 Interdisciplinary Center for Neurosciences, Heidelberg University, 69120 Heidelberg, Germany

**Keywords:** Alzheimer’s disease, Neurodegeneration, Insulin receptor, Gangliosides, Caveolin-1

## Abstract

Decreased neuronal insulin receptor (IR) signaling in Alzheimer’s disease is suggested to contribute to synaptic loss and neurodegeneration. This work shows that alteration of membrane microdomains increases IR levels and signaling, as well as neuronal viability in AD models in vitro and in vivo. Neuronal membrane microdomains are highly enriched in gangliosides. We found that inhibition of glucosylceramide synthase (GCS), the key enzyme of ganglioside biosynthesis, increases viability of cortical neurons in 5xFAD mice, as well as in cultured neurons exposed to oligomeric amyloid-β-derived diffusible ligands (ADDLs). We furthermore demonstrate a molecular mechanism explaining how gangliosides mediate ADDL-related toxic effects on IR of murine neurons. GCS inhibition increases the levels of functional dendritic IR on the neuronal surface by decreasing caveolin-1-mediated IR internalization. Consequently, IR signaling is increased in neurons exposed to ADDL stress. Thus, we propose that GCS inhibition constitutes a potential target for protecting neurons from ADDL-mediated neurotoxicity and insulin resistance in Alzheimer’s disease.

**Electronic supplementary material:**

The online version of this article (doi:10.1186/s40478-016-0354-z) contains supplementary material, which is available to authorized users.

## Introduction

Alzheimer’s disease is characterized by progressive neurodegeneration and loss of cognitive abilities. Typical histopathological hallmarks are the occurrence of extracellular amyloid-β (Aβ) plaques and intracellular neurofibrillary tangles [48]. Even though senile plaques containing Aβ fibrils accumulate in the brain as the disease progresses, soluble oligomeric Aβ species have been hypothesized to be the major neurotoxic agents in Alzheimer’s disease [31].

Accumulating evidence suggests that dysregulation of brain insulin receptor (IR) signaling is associated with the pathogenesis of Alzheimer’s disease [11, 15, 28, 57]. Signaling molecules downstream of the IR, including IR substrate (IRS)-1/2, PI3 kinase (PI3K) and phospho-Akt, are significantly down-regulated in the frontal cortex and hippocampus of Alzheimer’s disease patients and in Alzheimer’s disease mouse models [46]. Oligomeric amyloid-β-derived diffusible ligands (ADDLs) [22, 47] are spherical Aβ aggregates, with sizes ranging from 3 to 5 nm [31]. When hippocampal neurons in culture are exposed to ADDLs, dendritic IR are rapidly internalized from the neuronal cell surface [57]. Consequently, ADDL exposure results in impaired synaptic function and subsequent neurodegeneration [7, 16]. Increasing IR signaling has therefore emerged as a potential therapeutic target in Alzheimer’s disease [41].

Neuronal membrane lipid microdomains are highly enriched in glucosylceramide-synthase (GCS; gene: *Ugcg*)-derived gangliosides [21, 35]. Gangliosides directly modulate the activity of transmembrane receptors [21, 35]. Indeed, ganglioside expression is altered in Alzheimer’s disease [3]. Accumulation of gangliosides GM1 and GM2 was found in frontal and temporal cortex of Alzheimer’s disease patients [38], and GM1 has been proposed as a seed for aggregation and fibril formation of soluble Aβ [53]. Further studies involving transgenic mouse models of Alzheimer’s disease moreover suggest that gangliosides GQ1bα and GT1aα accumulate in the brains [2, 4]. However, mechanisms promoting ganglioside-related neurotoxicity in conjunction with Alzheimer’s disease have not yet been described. Thus, the present study has addressed the question whether GCS inhibition and subsequent ganglioside reduction might protect neurons in Alzheimer’s disease models in vitro and in vivo.

It has been hypothesized that ADDL-mediated toxicity to IR and neurons requires the presence of a heterologous complex involving additional, yet unknown membrane components [57]. Recently, we could show that GCS deletion increases IR sensitivity of hypothalamic neurons [21]. Indeed, we have found that GCS inhibition and subsequent reduction of gangliosides increases neuronal resistance towards Aβ stress by increasing levels of functional IR on the neuronal cell surface. Consequently, we have found less neurodegeneration in the cerebral cortex of 5xFAD mice [36] with GCS deletion in adult forebrain neurons. In line with this, pharmacological GCS inhibition by Genz-123346 (GENZ) increases cell viability in cultured neurons exposed to ADDLs. Furthermore we have now shown that GCS inhibition and subsequent ganglioside reduction decreases caveolin-1 levels and subsequent caveolae formation. This ultimately increases functional IR at the neuronal surface and specifically promotes insulin-dependent MAP kinase (MAPK, ERK1/2) signaling. Consequently, neuronal survival is enhanced in the applied Alzheimer’s disease models. Thus, we propose that GCS inhibition and subsequent ganglioside reduction constitute promising cellular targets for increasing insulin sensitivity in Alzheimer’s disease.

## Materials and methods

### Mice

Ugcgf/f//CamKCreERT2 mice were generated as described previously [35] and crossed to 5xFAD mice (The Jackson Laboratory) to generate 5xFAD//Ugcgf/f//CamKCreERT2 (5xFAD//Cre), 5xFAD//Ugcgf/f (5xFAD) and Ugcg f/f control littermates. Mice homozygous for the floxed *Ugcg* allele as well as heterozygous for the FAD mutations and Cre recombinase were used in all instances. All mice were backcrossed to the C57BL6 background at least 12 generations. Male mice were injected with tamoxifen 4 weeks after birth as described [35].

### Study approval

Animal experiments were approved by internal committees at the DKFZ Heidelberg and by Regierungspräsidium Karlsruhe (Germany).

### Brain sections and tissue samples

For morphological analysis, PLA, and ISH, brain hemispheres of mice were immersion-fixed in 4 % PFA (4 °C, 7 days) and subsequently embedded in paraffin according to standard procedures. 5 μM paraffin sections were prepared. Morphology was visualized by cresyl violet staining. Cortical layer 1 thickness was measured with Mirax Viewer software. The mean derived from four independent measurements per section was counted as *n* = 1 measurement. For biochemical analysis, tissue samples were freshly dissected and snap-frozen in liquid N_2_.

### In situ hybridization (ISH)

5 μM sagittal brain sections were prepared under RNase-free conditions. ISH was performed using a commercially available kit (RNAscope 2.0 HD Brown, Advanced Cell Diagnostics (ACD)) according to the manufacturer’s guidelines. Slides were exposed to either a probe recognizing Ugcg (ISH probe targeting region 653–1108 of mouse Ugcg mRNA (ACD)) or a negative control probe (ACD). Sections were subsequently counterstained with 50 % hematoxylin, immersed in a 70 %–100 % alcohol series as well as xylene, and finally mounted with Eukitt and coverslips. Slides were scanned with a digital slide scanner and analyzed with the Mirax Viewer software.

### Generation of ADDLs

ADDLs were prepared from monomeric human Aβ_1-42_ (Peptide Specialty Laboratories, Heidelberg) as described [47]. In brief, monomeric synthetic human Aβ_1-42_ was diluted in HFIP to acquire a concentration of 1 mM. Aliquots of this solution were freeze-dried overnight in a lyophilizer and stored at -20 °C until further use. Dried Aβ_1-42_ monomers were dissolved in DMSO, in order to acquire 5 mM solutions. In order to generate ADDLs, a 100 μM Aβ_1-42_ solution (DMEM) was generated, immediately mixed for 15 s, and incubated at 4 °C for 16 h. In order to generate proto-filaments, a 100 μM Aβ_1-42_ solution (DMEM) was incubated for 24 h at 4 °C. For the preparation of protofibrils and mature fibrils, the concentrated peptides were initially resuspended to 5 mM in DMSO, and then diluted with 10 mM HCl, resulting in a final concentration of 100 μM Aβ_1-42_. This solution was mixed for 15 s and incubated at 37 °C for 24 h and 48 h, in order to aggregate into protofibrils and mature fibrils, respectively. Aggregation states were confirmed by transmission electron microscopy and dot blot assays.

### Dot blot analysis of Aβ_1-42_ species

2 μl of the respective Aβ_1-42_ solution was applied onto nitro-cellulose membranes. The membranes were incubated with either oligomer-specific (A11, 1:200, Invitrogen) or Aβ_1-42_ –specific (4G8, 1:200, Covance) antibodies o/n. Membranes were then washed and blocked with 5 % skim milk/PBS for 1 h at RT. Secondary antibodies for dot blot were HRP-conjugated rabbit-anti-mouse (1:1000, DAKO) and HRP-conjugated goat-anti-rabbit (1:1000, DAKO). Spots were visualized by ECL (Amersham) and subsequent exposure to X-ray films.

### Cell culture

The mHippoE-14 cells were purchased from CELLutions Biosystems (Cedarlane, Canada) and cultured according to the respective manufacturer’s guidelines. Primary hippocampal neurons were generated and maintained as previously described [23] and used for experiments after 21 days in vitro. Cells were treated with GENZ, ADDLs, or insulin as indicated. Cell cultures were tested negative for mycoplasma.

### Immunofluorescence

Immunofluorescence of cells was performed as described by us earlier [21, 35]. Cultured neurons were grown and treated as indicated. Cells were immediately washed with ice-cold PBS and fixed in 4 % PFA (4 °C, 15 min). For surface staining of non-permeabilized cells, blocking and antibody incubations occurred in 1%BSA/PBS (RT, 1 h). For total staining of permeabilized cells, blocking and antibody incubations occurred in 1%BSA/0.05 % Triton-X/PBS (RT, 1 h). Primary antibodies were Alexa-Fluor488-conjugated α-Aβ (6E10; 1:200, Covance), rabbit-α-GM1 (1:20, Matreya), mouse-α-GD1a (1:50, Millipore), mouse-α-GT1b (1:50, Millipore), rabbit-α-IRα (1:50, Santa Cruz, N-20), goat-α-IRβ (1:50, Santa Cruz, D-17), rabbit-α-Cav-1 (1:50, Santa Cruz), mouse-α-synaptophysin (1:200, Millipore), goat-α-MAP2 (1:500, Millipore). Secondary antibodies were donkey-α-mouse Alexa-Fluor488, donkey-α-rabbit Alexa-Fluor488, goat-α-rabbit Alexa-Fluor546, goat-α-mouse Alexa-Fluor546, donkey-α-goat Alexa-Fluor546, and goat-α-mouse Alexa-Fluor633 (1:100, Invitrogen). Phalloidin staining was performed with phalloidin-Alexa-Fluor488 (1:200, Cell Signaling Technology), phalloidin-Alexa-Fluor546 (1:200, Cell Signaling Technology), and phalloidin-Alexa-Fluor633 (1:100, Cell Signaling Technology). Coverslips were mounted with ProLongGold® (Invitrogen) and subsequently analyzed by fluorescence (Keyence) or confocal microscopy (Leica).

### Cell viability assay

Cells were grown in 96-wells and treated as indicated. The ADDL incubation time was 24 h. 10 μl MTT solution was applied to each well and incubated at 37 °C for 4 h. MTT reduction products were then released from the cells by addition of 100 μl DMSO/well. Absorption was measured at 570 nm in an ELISA plate reader.

### Proximity ligation assay (PLA, Duolink®) of cultured neurons (surface or total PLA)

Cells were grown and treated as indicated. Cells were immediately washed with ice-cold PBS and fixed in 4 % PFA (4 °C, 15 min).For surface PLA, blocking and primary antibody incubations occurred in 1%BSA/PBS. For total PLA, blocking and incubations occurred in in 1 % BSA/0.05 % Triton-X/PBS. The PLA was performed according to the manufacturer’ s guidelines (Duolink®, Sigma). Primary antibodies were rabbit-α-IRα (1:50, Santa Cruz, N-20, extracellular epitope), goat-α-IRβ (1:50, Santa Cruz, D-17, extracellular epitope), mouse-α-Aβ (6E10; 1:100, BioLegend), mouse-α-GT1b (1:50, Millipore), mouse-α-GD1a (1:50, Millipore), rabbit-α-GM1 (1:50, Matreya), rabbit-α-Cav-1 (1:50, Santa Cruz). In case of ADDL/GD1a and ADDL/GT1b-PLA, the primary mouse-α-Aβ (6E10) was directly labeled for PLA with the PLA Probemaker® (Sigma), according to the manufacturer’s guidelines. Directly labeled ADDL PLA-PLUS probe was used at a 1:30 dilution. Primary antibodies were incubated at 4 °C o/n and PLA was subsequently performed according to the manufacturer’s guidelines (Duolink® Detection Reagents Orange or Green, Sigma). In the case of immortalized neurons, nuclei were stained with DAPI. After completion of PLA, phalloidin counterstaining was performed with phalloidin-Alexa-Fluor488 or phalloidin-Alexa-Fluor546 (1:200, Cell Signaling Technology, diluted in 0.01 % Triton-X/PBS), as indicated. PLA spots were visualized by fluorescence microscopy (Keyence) and quantified by ImageJ (NIH).

### Determination of IR phosphorylation by PLA on cultured neurons

Primary neurons were cultured in Neurobasal® medium (Gibco) and insulin-free B27 (Gibco). They were treated with 100nM insulin for 3 min. Cells were immediately washed with ice-cold PBS and fixed in 4 % PFA (4 °C, 15 min). Blocking and primary antibody incubations occurred in permeabilizing buffer (1%BSA/0.05 % Triton-X/PBS). For PLA analysis of IR tyrosine phosphorylation, primary antibodies were rabbit-α-IRβ (1:30, Santa Cruz, C-19, intracellular epitope) and mouse-α-phosphotyrosine clone 4G10® (1:50, Millipore). PLA was subsequently performed according to the manufacturer’s guidelines (Duolink® Detection Reagents Green, Sigma). After completion of PLA, phalloidin counterstaining was performed with phalloidin-Alexa-Fluor546 (1:200, Cell Signaling Technology, diluted in 0.01 % Triton-X/PBS). PLA spots along the dendrites were visualized by fluorescence microscopy (Keyence) and quantified by ImageJ (NIH).

### PLA of mouse brain sections

Mice were sacrificed, brains were immersion-fixed in PFA for 7 days and subsequently embedded in paraffin. 5 μM sections sections were prepared for PLA (Duolink®, Sigma). Prior to PLA, antigen retrieval was performed in a pressure heater (120 °C, 25 min) in citrate buffer (0.1 M C_6_H_9_Na_3_O_9_/0.1 M C_6_H_10_O_8_, pH 6.0). Blocking and primary antibody incubations occurred in 1%BSA/0.05 % Triton-X/PBS. Primary antibodies were α-IRα (1:30, Santa Cruz, N-20), α-IRβ (1:30, Santa Cruz, D-17), and mouse-α-Cav-1 (1:50, BD Biosciences). PLA was performed according to the manufacturer’ s guidelines as described above. Nuclei were stained with DAPI. PLA spots in cortex layer 5 neurons were visualized by fluorescence microscopy (Keyence). The number of spots in the vicinity of DAPI-stained nuclei was quantified.

### Determination of IR phosphorylation by co-immunoprecipitation (co-IP)

The mHippoE-14 cells were serum-starved for 4 h prior to stimulation with insulin for 3 min. Cells were then lysed and subjected to co-IP with agarose beads conjugated to α-IRβ-C19 (Santa Cruz), as previously described [21]. Subsequent SDS gel electrophoresis and Western blots were performed according to standard procedures [35]. Tyrosine phosphorylation of precipitated IR was visualized by mouse-α-phosphotyrosine clone 4G10® (1:200, Millipore). The secondary antibody was horseradish peroxidase–conjugated α-mouse IgG (1:1,000; Dako). Bands were visualized by chemiluminescence (Amersham) and quantified with ImageJ (National Institutes of Health).

### Co-IP of biotinylated ADDLs, and dot blot analysis of IR and GD1a

Biotinylated ADDLs were generated from commercially available biotinylated monomeric human Aβ_1-42_ (Peptide Specialty Laboratories, Heidelberg), as described for ADDLs above. The mHippoE-14 cells were either treated with saline or 5 μM biotinylated ADDLs, washed with ice-cold PBS, and lysed as described above. Protein levels were determined by Bradford (Sigma) and equal amounts of protein were subjected to Co-IP. Co-IP was performed with Dynabeads® M-270 streptavidin (Invitrogen) at 4 °C o/n. After washing and elution, 2 μl of each co-precipitated sample as well as the input lysate were loaded onto a nitrocellulose membrane for dot blot analysis. Dot blots were performed as described above. Primary antibodies were mouse-α-Aβ (4G8; 1:100, Covance), rabbit-α-IRβ (1:200, Santa Cruz), and mouse-α-GD1a (1:100, Millipore).

### Western blots

Brain tissue was immediately dissected and snap-frozen in liquid N_2_. Cultured cells were grown and treated as indicated. Lysates were prepared from tissue and cell cultures, as described by us earlier [35]. Protein concentrations were determined by Bradford assay (Sigma) and equal amounts of protein were loaded onto SDS gels. SDS gel electrophoresis and subsequent transfer to nitrocellulose membrane was performed according to standard procedures [35]. Primary antibodies used for western blot were rabbit-α-IRβ (1:200, Santa Cruz, C-19), rabbit-α-p35/p25 (1:500, Santa Cruz), rabbit-α-Cav-1 (1:1000, Santa Cruz), mouse-α-clathrin HC (1:200, Santa Cruz), mouse-α-synaptophysin (1:2000, Millipore), mouse-α-NMDAR1 (1:100, US Biological), rabbit-α-IRS-1 (1:100, Cell Signaling Technology), rabbit-α-IRS-2, rabbit-α-PI3 kinase p85, rabbit-α-Akt, rabbit-α-phospho-Akt, rabbit-α-GSK3β, rabbit-α-phospho-GSK3β, rabbit-α-phospho-ERK1/2, rabbit-α-β-actin (1:1000, Cell Signaling Technology), mouse-α-ERK1/2 (1:1000, BD Biosciences), rabbit-α-Grb2 (1:200, Santa Cruz), mouse-α-β-tubulin (1:500, Millipore). Secondary antibodies used were HRP-conjugated goat-α-rabbit (H + L) and HRP-conjugated rabbit-α-mouse (H + L) (1:1000, DAKO). Bands were visualized by chemiluminescence (Amersham) and quantified with ImageJ (National Institutes of Health). Bands were normalized to the respective loading controls.

### Surface biotinylation assay

Cells were treated with GENZ as indicated and surface proteins were biotinylated and subsequently isolated with help of a Surface Protein Isolation Kit (Pierce), according to the manufacturer’s guidelines. Surface proteins were separated by SDS-PAGE and subjected to the blotting procedure described above. IR bands were visualized with primary IR antibody (C-19, 1:200 in 5 % milk, Santa Cruz) and secondary HRP-conjugated anti-rabbit antibody (1:1000 in 5 % milk, DAKO).

### Transfections with siRNAs

The mHippoE-14 cells were seeded at a density of 10,000 per 6-well. The next day the medium was replaced by 2 ml fresh DMEM. Cells were transfected with either in total 3 nM control siRNA (Qiagen) or Cav-1 siRNA (Qiagen) for 7 days. Then, cells were processed for further analysis.

### Quantification of caveolae by EM

The mHippoE-14 cells were grown on coverslips and treated as indicated. Cells were then fixed in 2.5 % glutaraldehyde/ 0.05 M cacodylate buffer (RT, 10 min), followed by a second fixation step in 1.5 % osmium tetroxide. Ultrathin sections (70 nm) were prepared and stained with lead citrate and uranyl acetate. Cells were observed under an electron microscope (EM910, Zeiss) and the number of cell surface caveolae along the whole membrane per cell cross-section was counted for 10 cells per group.

### Quantitative mRNA analysis

Total RNA of the control and GENZ-treated mHippoE-14 cells was extracted and processed for qPCR Light Cycler (Roche) analysis as described earlier [35]. Expression levels were normalized to the housekeeping gene tubulin. The following primers were used: IR forward: 5’-GGAAC-CTAATGGTCTGATTGTGCT-3’; IR reverse: 5’-CGGACTCGAACACTGTAG-TTTCCT-3’; Tubulin forward: 5’-TCTCTCACCCTCGCCTTCTA-3’; Tubulin reverse: 5’-GGGTTCCAGGTCTACGAACA-3’.

### Thin layer chromatography (TLC)

Neurons were cultured and treated as indicated. Gangliosides and sphingomyelin were extracted, purified, and visualized by thin layer chromatography [35]. Ganglioside bands were visualized with 0.2 % orcinol in 10 % sulphuric acid at 120 °C for 10 min. Sphingomyelin was visualized with CuSO4 in 8 % H3PO4 at 180 °C for 10 min.

### Immune overlay TLC

Immune overlay TLC was performed as described earlier by us [35]. In brief, gangliosides were extracted, purified, and separated on HPTLC silica gel plates as described above. HPTLC plates were then immersed in a solution composed of 0.5 % plexigum/chloroform diluted 1:10 in n-hexan for 2 min. Plates were allowed to dry afterwards. After immersion in blocking solution (1 % BSA in PBS; RT, 1 h) plates were incubated with primary antibodies at 4 °C o/n. Primary antibodies were rabbit-α-GM1 (1:100, Matreya), mouse-α-GD1a (1:500, Millipore), mouse-α-GT1b (1:500, Millipore), and mouse-α-GM3 (IgM) (1:250, Wako). Secondary antibodies were alkaline phosphatase-conjugated goat-α-rabbit (H + L) or alkaline phosphatase-conjugated goat-α-mouse (H + L) (1:500, Jackson Immunoresearch). The AP signal was visualized with SigmaFastTM (Sigma Aldrich). For subsequent visualization of all ganglioside-containing bands, the HPTLC plate was rinsed with H_2_O and acetone. Bands were subsequently visualized with 0.2 % orcinol in 10 % sulphuric acid at 120 °C for 10 min.

### Statistics

Data are presented as mean ± SEM. Statistical analysis was done with Graph Pad Prism. Comparison of mean values from two groups were performed by an unpaired two-tailed Student’s *t*-test. Values were considered as significant if *p* ≤ 0.05 and marked with (*). Results were marked with (**) if *p* ≤ 0.01, or (***) if *p* ≤ 0.001.

## Results

### Inhibition of GCS-mediated ganglioside biosynthesis by GENZ increases resistance towards ADDLs and IR signaling in mHippoE-14 neurons

**Fig. 1:**
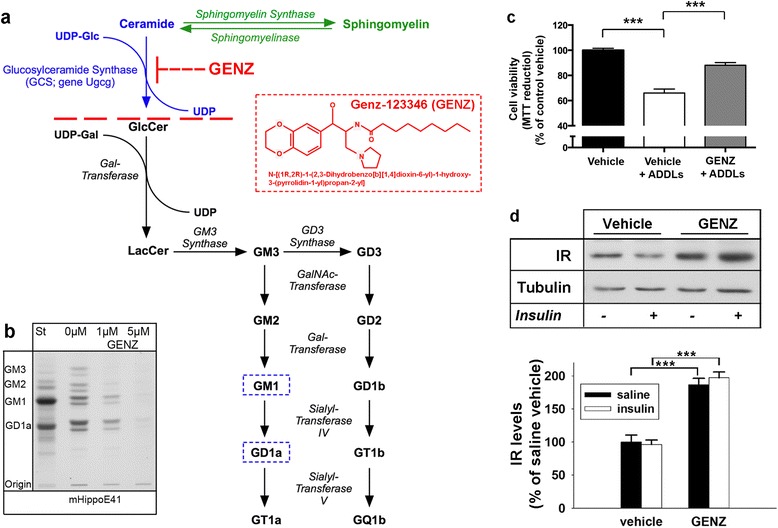
GCS inhibition by GENZ-123346 leads to increased viability of hippocampal mHippoE-14 neurons exposed to Aβ_1-42_-derived diffusible ligands (ADDLs). **a** General ganglioside biosynthesis and the neuronal a-series gangliosides expressed in mHippoE-14 cells (outlined). Pharmacological inhibition of the key enzyme involved in ganglioside biosynthesis, glucosylceramide synthase (GCS) by the ceramide analogue Genz123346 (GENZ) abates ganglioside biosynthesis. **b** Thin layer chromatography (TLC) shows that a concentration of 5 μM GENZ (7 days) efficiently inhibits ganglioside biosynthesis in mHippoE-14 cells. **c** An MTT assay confirms the toxicity of the ADDLs (white bar). However, GENZ treatment increases neuronal viability upon ADDL stress (grey bar; *n* = 5-12 replicates). **d** Western blot shows that total insulin receptor (IR) levels are increased in GENZ-treated mHippoE-14 cells (*n* = 4). Unpaired two-tailed student’s *t*-test (*p* ≤ 0.001 is marked with (***)); 5 μM ADDLs, 24 h; 100nM insulin, 10 min. Means ± SEM

Major neuronal gangliosides GM1, GD1a, GD1b, and GT1b are generated by the sequential addition of carbohydrate moieties to glucosylceramide (Fig. 1a). Thin layer chromatography (TLC) shows that the mouse hippocampal cell line mHippoE-14 expresses high levels of the a-series gangliosides GM1 and GD1a (Fig. 1b and Additional file 1: Figure S1a). The key enzyme in ganglioside biosynthesis, GCS, can be inhibited pharmacologically by GENZ (Fig. 1a). We found that treatment with 5 μM GENZ for 7 days efficiently inhibited GCS activity and subsequent ganglioside biosynthesis (Fig. 1b). GENZ-treated mHippoE-14 cells displayed an overall cell morphology resembling control cells, an unchanged synaptophysin expression, as well as unaltered cell viability (Additional file 1: Figure S1b, c, and d).

In order to mimic Aβ stress in vitro, we generated oligomeric ADDLs by defined incubation and aggregation of synthetic Aβ_1-42_ [47], which exert neurotoxicity [31, 32]. The successful generation of oligomeric ADDLs was verified by electron microscopy and a dot blot using the oligomer-specific antibody A11 (Additional file 1: Figure S2a and b). The generated ADDLs bound to mHippoE-14 cells (Additional file 1: Figure S2c). An MTT assay furthermore confirmed the toxicity of the generated ADDLs, since cell viability decreased in mHippoE-14 cells exposed to 5 μM ADDLs (Fig. 1c, white bar). This concentration of ADDLs has furthermore been proven useful for immortalized cell lines by other groups [8, 31]. Importantly, however, mHippoE-14 cells pre-treated with GENZ were more resistant towards ADDL stress (Fig. 1c, grey bar).

Previous studies showed that ADDLs are hypothesized to exert neurotoxic effects by directly interfering with synaptic integrity [30] and, more specifically, by decreasing neuronal IR levels and IR signaling [11, 15, 28]. We have previously reported that genetic GCS deletion increased IR levels in hypothalamic neurons of mice [21]. In line with this, a western blot revealed that pharmacological GCS inhibition by GENZ was also able to increase total IR levels in vitro (Fig. 1d). Furthermore, the observed elevation of IR levels by GENZ occurred independently of insulin stimulation.

**Fig. 2:**
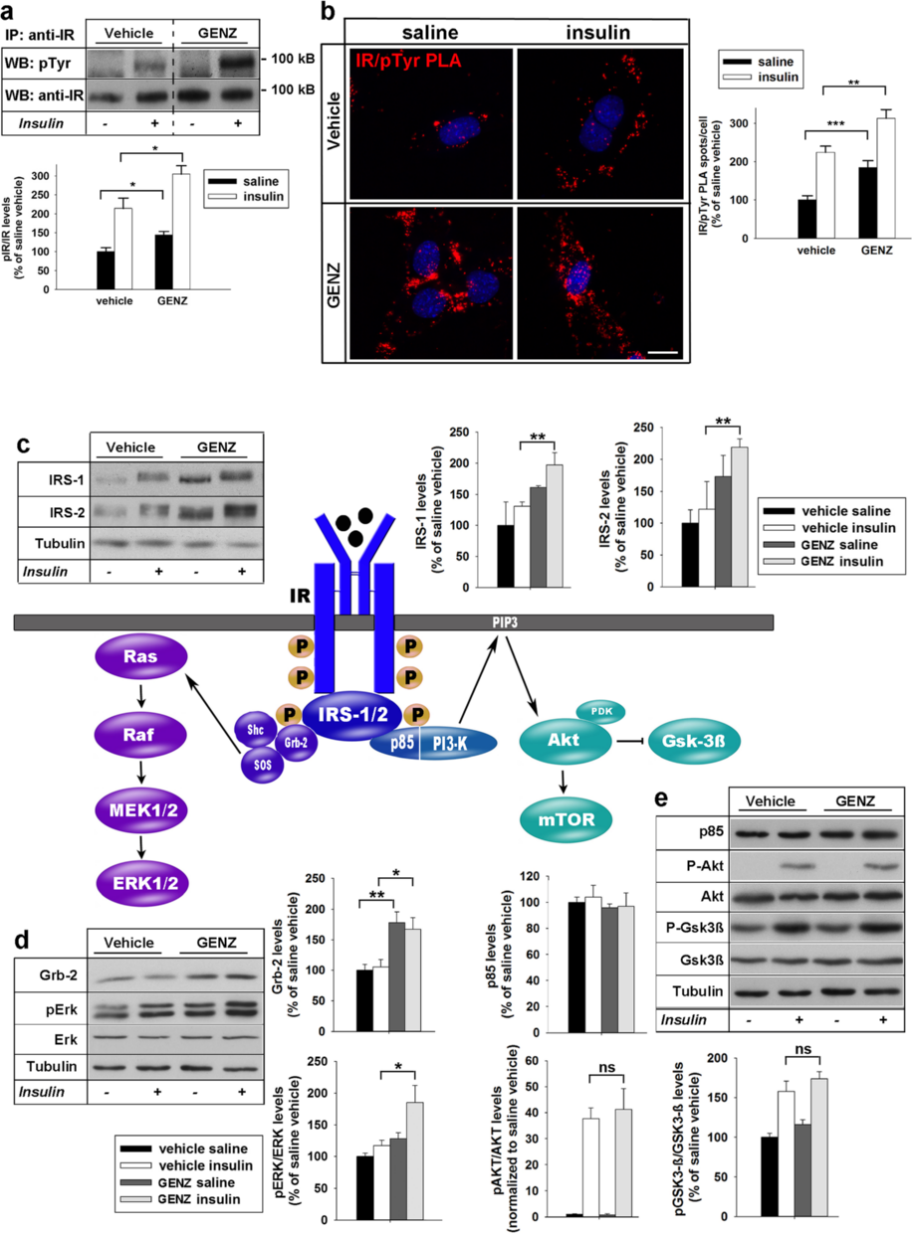
GENZ treatment increases IR signaling in mHippoE-14 neurons. **a** GENZ treatment increases IR phosphorylation, as shown by IR-co-IP and subsequent phospho-tyrosine staining (*n* = 4). Bands of vehicle- and GENZ-treated mHippoE-14 cells were derived from the same membrane and the same film exposure. **b** Proximity ligation assay (PLA) confirms that GENZ treatment enhances insulin-dependent IR tyrosine phosphorylation (IR/pTyr; *n* = 49–69 cells). **c** IRS-1 and IRS-2 levels are increased in GENZ-treated cells (*n* = 4). **d** Grb-2 and p-ERK/ERK are increased in GENZ-treated cells (Grb-2: *n* = 4, pERK/ERK: *n* = 8). **e** p85, p-AKT, AKT, p-Gsk3β, and Gsk3β are not changed by GENZ treatment (*n* = 4). Cells were treated with either saline or 100 nM insulin for 3 min ((**a**) – (**b**)) or 10 min ((**c**) – (**e**)). Unpaired two-tailed student’s *t*-test (if *p* ≤ 0.05, *p* ≤ 0.01, or *p* ≤ 0.001 results are marked with (*),(**) or (***), respectively). Means ± SEM. Scale bar: 10 μm

We next evaluated if GENZ treatment also increased insulin-evoked IR signal transduction in mHippoE-14 cells. Indeed, we observed more prominent IR phosphorylation in GENZ-treated neurons, as shown by IR/pTyr co-IP (Fig. 2a). A PLA directly showing phosphorylated IR in mHippoE-14 cells (IR/p-Tyr) confirmed that GENZ treatment equally increases IR phosphorylation compared to control cells upon stimulation with two different insulin concentrations: 100 nM [16, 54] (Fig. 2b) and 10 nM [19] (Additional file 1: Figure S3a, b). An analysis of IR downstream signaling subsequently revealed that IRS-1-, IRS-2-, and Grb-2 levels were also significantly increased in cells pre-treated with GENZ (Fig. 2c and d). We found that GENZ treatment specifically enforced insulin-stimulated transduction via the MAP kinase pathway, as Erk phosphorylation was significantly enhanced (Fig. 2d). However, GENZ treatment did not increase p85 or insulin-dependent phosphorylation of Akt and Gsk3β in mHippo-E14 cells (Fig. 2e).

These results indicate that mHippoE-14 cells treated with GENZ exhibit normal viability. In addition, GCS inhibition and subsequent reduction of gangliosides lead to an increased neuronal resistance towards ADDLs. Furthermore, GENZ-mediated ganglioside reduction increases the levels of functional neuronal IR and subsequent MAPK signaling.

### Pharmacological GCS inhibition elevates IR levels on the surface of ADDL-treated mHippoE-14 neurons by decreasing Cav-1 expression

**Fig. 3:**
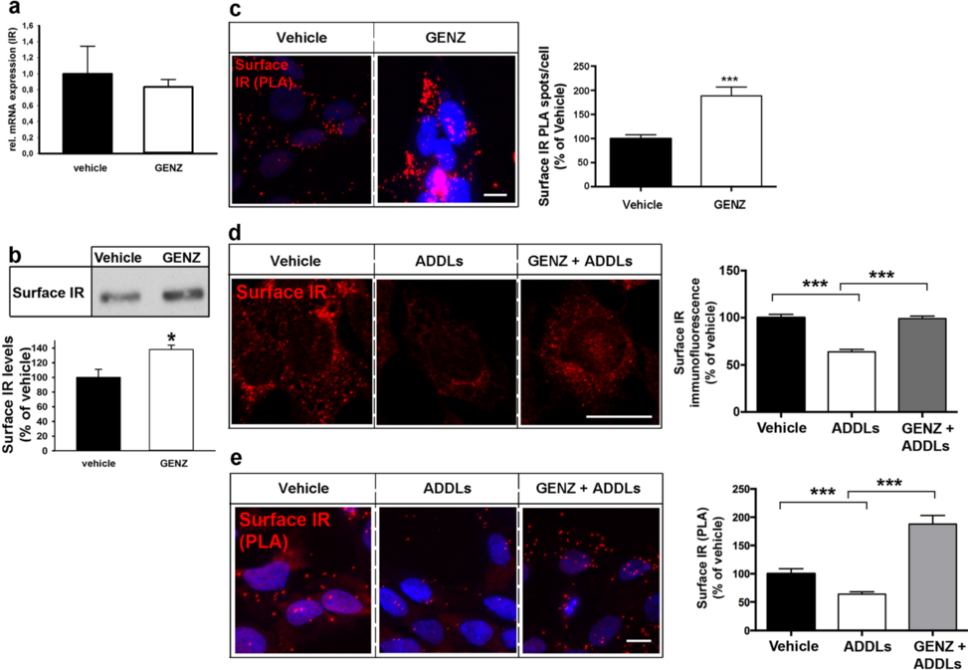
GCS inhibition by GENZ increases IR on the surface of mHippoE-14 neurons exposed to ADDLs. **a** Quantitative PCR shows that IR mRNA is not changed by GCS inhibition. **b** A surface biotinylation assay and subsequent western blot analysis of the precipitated IR show GENZ-dependent sequestering of the IR to the cell surface (*n* = 5). **c** A PLA using two different IR antibodies depicts surface IR levels on non-permeabilized cells. GENZ treatment increases surface IR levels (*n* = 97–135 cells). **d** Immune fluorescence shows surface IR of non-permeabilized cells. ADDL exposure decreases surface IR levels (white bar). However, surface IR levels are increased in cells treated with GENZ (grey bar; *n* = 121–212 cells). **e** A PLA on non-permeabilized cells using two IR antibodies (N-20 and D-17) confirms that GENZ treatment increases surface IR upon ADDL exposure (*n* = 119–186 cells). Unpaired two-tailed student’s *t*-test (if *p* ≤ 0.05, or *p* ≤ 0.001 results are marked with (*) or (***), respectively); 5 μM ADDLs, 24 h. Means ± SEM. Scale bars: 10 μm

An initial evaluation revealed that IR mRNA expression was unaltered (Fig. 3a). Thus, the increase in IR levels of GENZ-treated cells could not be explained by increased transcription. Importantly, total levels of NMDA receptor, another receptor abundantly located at synapses, were not changed by GENZ treatment (Additional file 1: Figure S4a). This rules out a generalized, non-specific effect of GENZ on membrane receptor homeostasis.

However, both a surface biotinylation assay (Fig. 3b) as well as a proximity ligation assay on non-permeabilized cells (Fig. 3c and Additional file 1: Figure S4b) suggested that IR levels were increased at the cellular surface of GENZ-treated cells. In order to pinpoint this effect to the loss of gangliosides in GENZ-treated cells, we additionally analyzed cells treated with a different GCS inhibitor, namely the iminosugar n-butyldeoxynojirimycin (NB-DNJ) [39]. NB-DNJ treatment resulted in a ganglioside reduction of between 30 and 50 % (Additional file 1: Figure S4c) and also stabilized the levels of surface IR on mHippoE-14 cells exposed to ADDLs (Additional file 1: Figure S4d). Thus, we conclude that the loss of gangliosides increases the levels of surface IR in neurons independent of the chemical nature of inhibition.

ADDLs are hypothesized to exert major neurotoxic effects by eliciting the removal of IR from the neuronal surface [11, 15, 28]. Consequently, we next investigated if gangliosides are involved in this process. We found that 24 h ADDL exposure specifically decreased surface IR levels, as shown by surface immunofluorescence of non-permeabilized mHippoE-14 cells (Fig. 3d, white bar). However, ADDLs had only minor impact on total cellular IR levels (Additional file 1: Figure S5a). Loss of surface IR was additionally confirmed by proximity ligation on non-permeabilized cells (Fig. 3e, white bar, and Additional file 1: Figure S5b). Remarkably, however, pre-treatment with GENZ increased surface IR on ADDL-exposed neurons (Fig. 3d and e, grey bars).

**Fig. 4:**
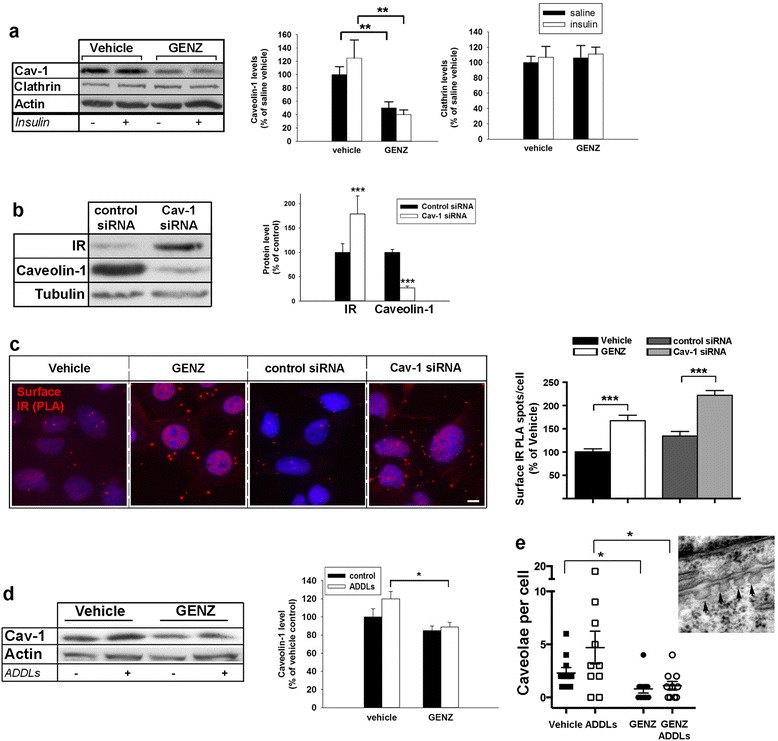
Surface IR are increased on GENZ-treated mHippoE-14 cells due to decreased levels of caveolin-1. **a** Western blot shows that total caveolin-1 but not clathrin levels are decreased in GENZ-treated cells (100 nM insulin, 10 min, *n* = 4). **b** Cells are either treated with caveolin-1- or control siRNA. Western blot confirms that the siRNA efficiently reduces caveolin-1. Total IR levels are increased in caveolin-1 siRNA treated-cells (*n* = 4). **c** A PLA shows that treatment with either caveolin-1 siRNA or GENZ increases surface IR on non-permeabilized cells (*n* = 91–129 cells). **d** Caveolin-1 is decreased in GENZ-treated mHippoE-14 cells despite ADDL exposure (*n* = 4). **e** The number of caveolae has been evaluated by EM of vehicle- or GENZ-treated cells exposed to either saline or ADDLs. GENZ treatment decreases the number of caveolae despite ADDL exposure. The typical morphology of structures defined as caveolae is shown in the inset (arrowheads). Cells with a completely intact membrane have been chosen and the total number of caveolae per cell membrane outline has been counted (*n* = 10 cells). Unpaired two-tailed student’s *t*-test (if *p* ≤ 0.05, *p* ≤ 0.01, or *p* ≤ 0.001 results are marked with (*), (**) or (***), respectively); 5 μM ADDLs, 24 h. Means ± SEM. Scale bar: 5 μm

In order to reveal a potential mechanism as to how membrane microdomains containing gangliosides may regulate surface IR in Alzheimer’s disease, we studied the process of IR endocytosis involving caveolin-1. Increases in caveolin-1 levels have been reported in Alzheimer’s disease previously [20], supporting a potential role of caveolin-1 in ADDL toxicity. GENZ treatment decreased overall caveolin-1 levels, whereas clathrin expression remained unchanged (Fig. 4a). In order to determine if caveolin-1 reduction by itself alters IR levels, we treated neurons with siRNA targeting caveolin-1 (Cav-1 siRNA). Strikingly, a marked reduction in caveolin-1 itself could mimic the increase in both total IR (Fig. 4b) and surface IR (Fig. 4c), which were initially observed in GENZ-treated cells.

Moreover, caveolin-1 levels were indeed lower in GENZ-treated mHippoE-14 cells, despite exposure to ADDLs (Fig. 4d). We next investigated if the reduction in caveolin-1 was also reflected by less caveolae formation. Therefore, we assessed the number of caveolae by an electron-microscopic approach. Corresponding to the lower caveolin-1 levels, caveolae were reduced in GENZ-treated mHippoE-14 cells exposed to ADDLs, when compared to ADDL-exposed cells that were not treated with GENZ (Fig. 4e).

We have previously observed that GCS deletion increases sphingomyelin levels [23, 35], which was also found in GENZ-treated neurons (Additional file 1: Figure S6a). However, GCS deletion does not change the levels of ceramide [23, 35]. We furthermore observed that sphingomyelin levels were not altered in Cav-1-siRNA-treated cells (Additional file 1: Figure S6b).

These results lead to the conclusion that membrane gangliosides facilitate the ADDL-induced IR removal from the surface. We surmise that surface IR levels are increased in GENZ-treated cells due to a reduction in caveolin-1 levels and caveolae formation indicative for caveolin-mediated endocytosis, which may be attributed to the loss of gangliosides.

### GENZ treatment prevents acute ADDL-mediated complex formation between the IR and ganglioside GD1a as well as caveolin-1

**Fig. 5:**
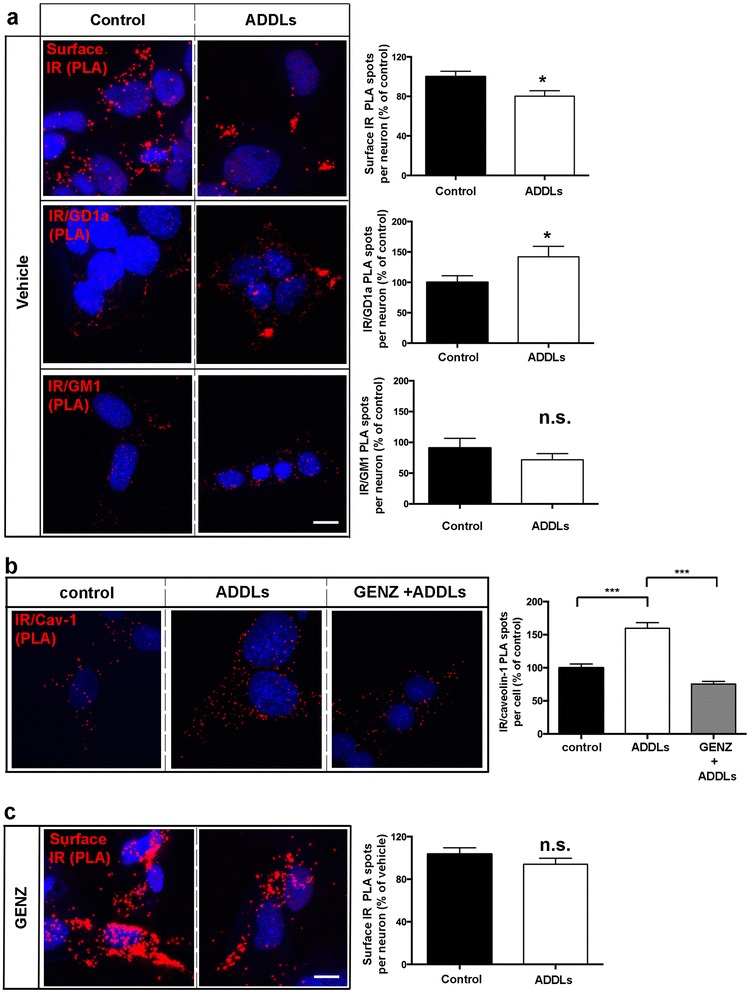
Neuronal gangliosides mediate acute ADDL-induced interaction of IR and caveolin-1 and subsequent loss of surface IR in mHippoE-14 neurons. **a** PLAs have been performed on non-permeabilized cells. Acute ADDL exposure leads to loss of IR from the cellular surface (upper graph). Simultaneously, ADDLs induce complex formation between IR and ganglioside GD1a (middle graph). However, ADDLs do not induce IR/GM1 interactions (lower graph; surface IR: *n* = 150–170 cells, IR/GD1a: *n* = 42–46 cells, IR/GM1: *n* = 27–30 cells). **b** The PLA shows that ADDLs stimulate complex formation between IR and caveolin-1. Increased ADDL-induced complex formation is not evident in GENZ-treated cells (*n* = 66–94 cells). **c** A PLA using two IR antibodies (N-20 and D-17) shows that surface IR are not reduced on GENZ-treated cells that have been acutely exposed to ADDLs (*n* = 149–170 cells). Unpaired two-tailed student’s *t*-test (*p* ≤ 0.05 is marked with (*)); 5 μM ADDLs, 30 min. Scale bars: 10 μM

In order to provide more direct evidence for the suggestion that membrane gangliosides take part in ADDL-elicited IR removal from the surface, we investigated ADDL-exposed cells by proximity ligation. Congruent with our observations from long-term ADDL exposure, PLA showed that acute ADDL exposure also induced loss of surface IR from mHippoE-14 cells (Fig. 5a). At the same time, complex formation between the IR and GD1a was observed, whereas IR and GM1 did not show any comparable interaction dynamics (Fig. 5a). Specificity of the applied antibodies to their respective gangliosides was verified by immune overlay TLC on known standard mouse brain lysate (Additional file 1: Figure S7a).

Importantly, ADDL exposure stimulated complex formation between IR and caveolin-1 in mHippoE-14 cells, which was less abundant upon GENZ treatment (Fig. 5b). Consequently, surface IR levels upon acute ADDL exposure were not reduced in GENZ-treated cells (Fig. 5c).

These data indicate that membrane gangliosides, specifically GD1a, obviously facilitate the ADDL-induced complex formation between the IR and caveolin-1. We furthermore assume that gangliosides take part in acute ADDL-mediated IR internalization. This neurotoxic effect can thus be prevented by GENZ-mediated inhibition of GCS.

### GCS inhibition increases IR levels on primary hippocampal dendrites

We next investigated if ganglioside-deficient primary neurons also exert increased resistance towards ADDL toxicity.

**Fig. 6:**
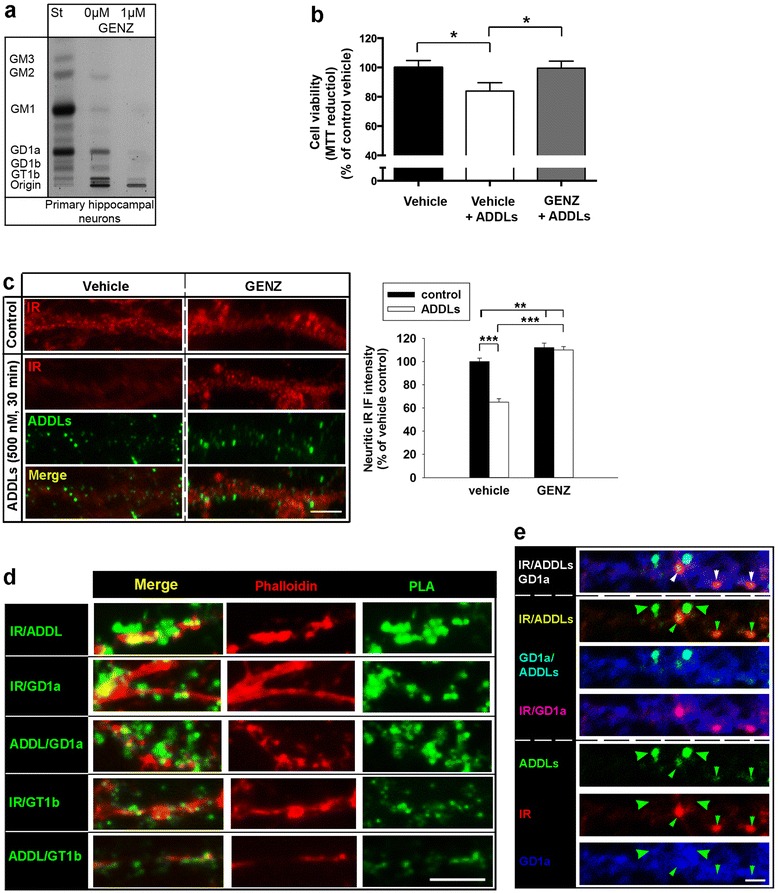
GCS inhibition also protects primary hippocampal neurons upon ADDL exposure. **a** A TLC shows that a concentration of 1 μM GENZ (3 days) efficiently inhibits ganglioside biosynthesis in primary mouse hippocampal neurons. **b** Exposure to ADDLs decreases the viability of neurons, as determined by an MTT assay (*white bar*). However, GENZ treatment of primary neurons maintains viability upon ADDL stress (*grey bar*; 1 μM ADDLs, 24 h; *n* = 12 replicates). **c** Surface immunofluorescence of dendritic IR and ADDLs (antibody 6E10) on a non-permeabilized primary neuron (1 μM ADDLs, 30 min). The fluorescence intensity of IR staining has been measured along the dendrites (*n* = 169–285 measurements). ADDL exposure leads to IR removal from the dendrite, whereas dendritic IR are increased in GENZ-treated neurons. **d** Combined PLA/phalloidin staining shows the respective PLA complexes (*green labels*) on primary dendrites. IR interact with ADDLs and gangliosides GD1a and GT1b. ADDLs themselves are in close proximity of GD1a and GT1b. **e** Triple immunofluorescence directly visualizes the proposed IR/ADDL/GD1a complexes (white arrowheads). Areas with strong ADDL binding co-label with GD1a, but IR staining is weak due to IR removal (big green arrowheads). However, IR/ADDL/GD1a complexes are found at locations with weaker ADDL staining (small arrowheads). Unpaired two-tailed student’s *t*-test (if *p* ≤ 0.05, *p* ≤ 0.01, or *p* ≤ 0.001 results are marked with (*), (**) or (***), respectively); 1 μM ADDLs, 24 h. Scale bars: 5 μM

Cultures of primary hippocampal neurons expressed the major neuronal a- and b-series of gangliosides GM1, GD1a, GD1b and GT1b. In order to inhibit ganglioside biosynthesis, we treated these neurons with GENZ. Treatment with 1 μM GENZ for 3 days efficiently inhibited ganglioside biosynthesis in primary neurons (Fig. 6a). We next studied if GENZ had any effect on the morphology of the dendrites. We found that GENZ-treated primary neurons displayed neuronal processes with spines and immunohistochemically visible pre- and postsynaptic contacts, as indicated by proximity of synaptophysin and phalloidin [45], respectively (Additional file 1: Figure S7b). Exposure to 1 μM ADDLs has been moreover proven efficient in primary neurons by others [17]. In congruence with mHippoE-14 cells, GENZ-treated primary neurons displayed increased resistance towards ADDL stress (1 μM ADDLs, 24 h), as shown in a cell viability assay (Fig. 6b). As it has been reported previously [16], ADDL exposure elicited IR removal from hippocampal dendrites (Fig. 6c). Remarkably and consistent with our results obtained in mHippoE-14 cells, pre-treatment with GENZ also increased surface IR levels on ADDL-exposed primary neurons (Fig. 6c).

These results indicate that GENZ treatment also protects primary hippocampal neurons from ADDL stress.

### Gangliosides take part in complex formation between IR, caveolin-1, and ADDLs along the dendrites and facilitate IR desensitization by ADDLs

It has been proposed that ADDL-mediated toxicity on IR requires the presence of a heterologous complex involving further membrane components [57]. Dendritic IR of GENZ-treated primary neurons were preserved, even though ADDL binding itself was unaltered (Fig. 6c). Consequently, we have investigated if gangliosides are involved in complex formation between ADDLs and dendritic IR, since this might be a prerequisite for mediating toxic ADDL effects on IR signaling. A co-labeling confirmed that ADDLs co-localize with dendritic IR (Additional file 1: Figure S7c). Immune fluorescence further indicated that ADDLs bound to dendrites in part co-localized with GD1a and GT1b (Additional file 1: Figure S7d, e, and f). GD1a and GT1b themselves also partially co-localized with dendritic IR (Additional file 1: Figure S7e and f). Surprisingly, however, very little interaction between GM1 and ADDLs could be observed (Additional file 1: Figure S7g). These results were confirmed by combined PLA/phalloidin stainings, which showed complex formation of GD1a and GT1b with both bound ADDLs and IR at dendrites (Fig. 6d and Additional file 1: Figure S7h). Moreover, PLA confirmed the sparse co-localization of GM1 with IR as well as with ADDLs (Additional file 1: Figure S7i).

In order to directly visualize the ADDL/IR/GD1a complex formation, we additionally performed a triple staining. As prominent ADDL binding ultimately leads to loss of surface IR, these complexes could be verified at sites with relatively low ADDL presence (Fig. 6e, white arrowheads). Additionally, our hypothesis of complex formation between biotinylated ADDLs, IR, and GD1a was supported by streptavidin co-immunoprecipitation (co-IP) and subsequent dot blot assays (Additional file 1: Figure S7j).

**Fig. 7:**
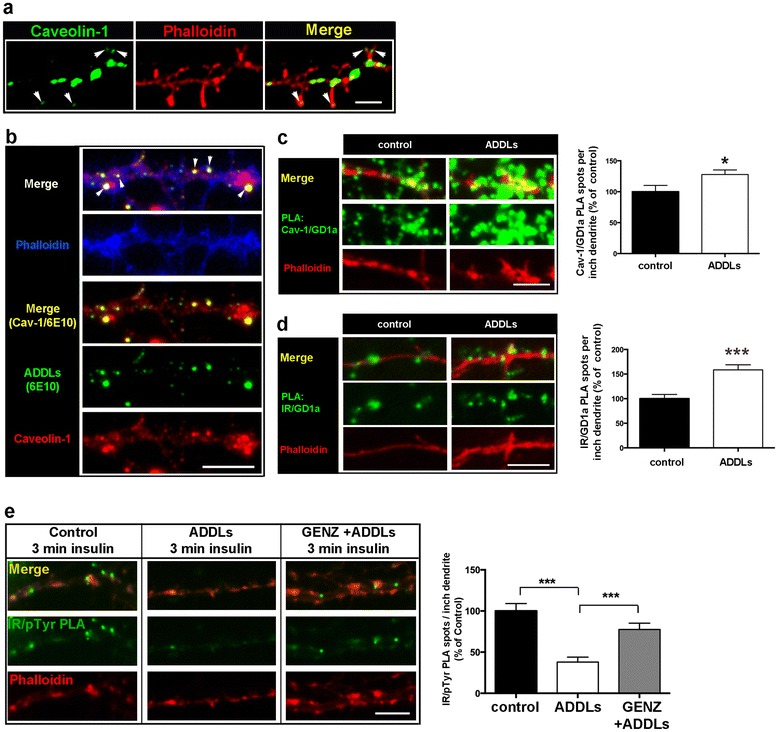
Acute ADDL-induced complex formation between IR, caveolin-1 and GD1a coincides with decreased IR phosphorylation at dendrites of primary hippocampal neurons. **a** Immunofluorescence depicts caveolin-1 localization at dendrites. **b** ADDLs bind to caveolin-1 at dendrites. **c** – **d** PLA shows that ADDLs stimulate (**c**) caveolin-1/GD1a and (**d**) IR/GD1a complex formation at dendrites. Quantification shows PLA spots/inch dendrite ((**c**) *n* = 46–50 measurements; (**d**) *n* = 29–44 measurements from 6–8 independent high power fields). **e** A PLA using both an IR- and a phospho-tyrosine-specific antibody indicates insulin-evoked dendritic IR phosphorylation (IR/pTyr; green). ADDL exposure decreases IR phosphorylation (white bar). However, GENZ treatment increases insulin sensitivity of dendritic IR upon ADDL exposure (grey bar). Quantification shows PLA spots/inch dendrite (*n* = 9–13 measurements). Cells were treated with either saline or 100nM insulin for 3 min. Dendrites were visualized with phalloidin. Unpaired two-tailed student’s *t*-test (if *p* ≤ 0.05, *p* ≤ 0.01, or *p* ≤ 0.001 results are marked with (*), (**) or (***), respectively); 1 μM ADDLs, 30 min. Scale bars: 5 μM

Caveolin-1, an important mediator of the endocytosis of dendritic IR, can be found along the dendrite and at dendritic spines (Fig. 7a, white arrowheads). A triple-labeling approach showed that ADDL staining overlaps in part with caveolin-1 localization at hippocampal dendrites (Fig. 7b), which supports a potential role of caveolin-1 in ADDL toxicity. Indeed, we found that acute ADDL exposure reduced dendritic surface IR (Fig. 6c), while simultaneously increasing caveolin-1/GD1a (Fig. 7c and Additional file 1: Figure S7k) as well as IR/GD1a (Fig. 7d and Additional file 1: Figure S7k) complex formation along the dendrites.

Consequently, we next analyzed if inhibition of ganglioside biosynthesis by GENZ might also prevent the ADDL-induced desensitization of dendritic IR. A PLA directly visualized phosphorylated IR (IR/p-Tyr) on dendrites. The PLA showed that ADDL exposure equally decreased IR phosphorylation upon stimulation with either 100 nM (Fig. 7e, white bar, and Additional file 1: Figure S8a) or 10 nM insulin (Additional file 1: Figure S8b). However, IR of neurons pre-treated with GENZ indeed retained insulin sensitivity when they were exposed to ADDLs (Fig. 7e and Additional file 1: Figure S8b, grey bars).

These results provide evidence that gangliosides also facilitate IR removal from the dendritic surface of primary neurons. The data furthermore suggest that complex formation between dendritic IR and ADDLs takes place in membrane microdomains enriched in GD1a. Furthermore, ganglioside GT1b is also hypothesized to take part in complex formation between IR and ADDLs. Importantly, GENZ-mediated ganglioside depletion prevents ADDL-induced desensitization of dendritic IR of primary hippocampal neurons.

### Cortical neurons of 5xFAD mice with genetic GCS deletion are more resistant towards ADDL toxicity

**Fig. 8:**
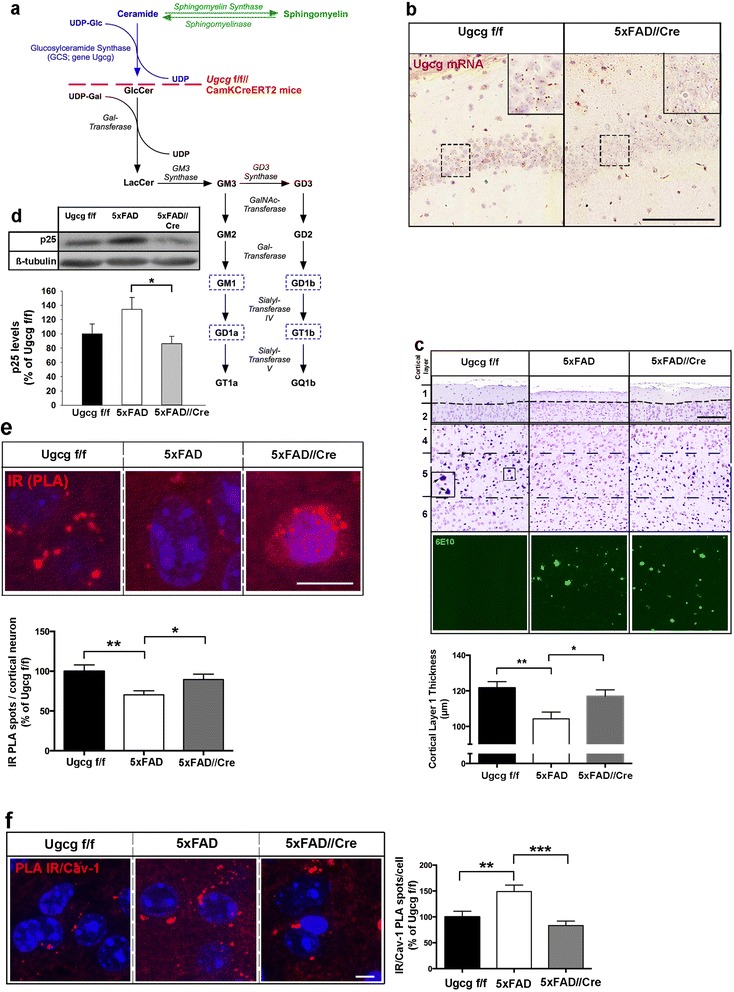
Genetic GCS inhibition increases neuronal viability in a 5xFAD mouse model of Alzheimer’s disease. **a** The biosynthesis of the major neuronal a- and b-series gangliosides (outlined) is inhibited by Cre-mediated deletion of GCS under the inducible forebrain-specific CamKIIα promoter. **b** In situ hybridization shows that Ugcg mRNA is almost completely absent in hippocampal CA1 region of 5xFAD//Cre mice (scale bar: 200 μM). **c** Cresyl violet staining of cortical layers of 7 months old mice. A layer 5 pyramidal neuron is depicted (inset, arrowheads). Layer 1 thickness is reduced in 5xFAD mice, but maintained in 5xFAD//Cre mice (*n* = 21–27 measurements from 8–11 mice). Aβ plaques in cortical layer 5 are stained by the antibody 6E10. **d** Western blot of the neurodegeneration marker p25 indicates neurodegeneration in 5xFAD mice, which is less pronounced in 5xFAD//Cre mice (*n* = 8–10 mice). **e** Total IR in cortical neurons have been quantified by PLA using two different IR antibodies (N-20 and D-17) in 7 months old Ugcgf/f, 5xFAD, and 5xFAD//Cre mice. The PLA shows that the decrease in IR in 5xFAD mice is less pronounced in 5xFAD//Cre mice (*n* = 80–98 cells from 3–5 mice per group, scale bar: 10 μM). **f** PLA shows that IR/Cav-1 proximity is increased in cortical neurons of 7 months old 5xFAD mice, and comparable to controls in 5xFAD//Cre mice (*n* = 71–103 neurons from 3–5 mice per group, scale bar: 5 μM). Unpaired two-tailed student’s *t*-test (if *p* ≤ 0.05, *p* ≤ 0.01 or *p* ≤ 0.001, results are marked with (*), (**), or (***), respectively). Means ± SEM

In order to find out if gangliosides play a role in Aβ toxicity in vivo, *Ugcg*f/f//CamKCreERT2 mice [35] were bred to 5xFAD//*Ugcg*f/f mice (5xFAD mice) harboring five familial AD mutations (APP K670N/M671L (Swedish mutation), I716V (Florida mutation), V717I (London mutation), and presenilin1 M146L and L286V) [36]. This results in an Alzheimer’s disease mouse model where GCS can be deleted specifically in adult forebrain neurons by tamoxifen injection (5xFAD//Cre mice) (Fig. 8a and Additional file 1: Figure S9a). An *in situ* hybridization of the hippocampal CA1 region showed that *Ugcg* mRNA was almost completely absent in 5xFAD//Cre mice (Fig. 8b).

A morphologic examination of neuronal integrity in 7 months old mice was carried out as described earlier for this mouse model [36]. It revealed that 5xFAD mice lost a substantial part of cortical layer 1 (Fig. 8c, white bar), which confirms the findings reported earlier [36]. This loss of layer 1 thickness is regarded to proportionally reflect the loss of pyramidal neurons in cortical layer 5, as pyramidal neurons of layer 5 project to and ramify in layer 1 [36]. Interestingly, layer 1 thickness was preserved in 5xFAD//Cre mice (Fig. 8c, grey bar), thus demonstrating that pyramidal neurons in cortical layer 5 of 5xFAD//Cre mice were protected. Remarkably, however, Aβ plaque load of 5xFAD//Cre mice was not decreased compared to 5xFAD mice (Fig. 8c). Decreased levels of p25, a marker indicative for neurodegeneration [36, 37] in cerebral cortex of 5xFAD//Cre mice further confirmed that their neurons were protected from Aβ stress (Fig. 8d).

While IR levels were lower in cortical neurons of 5xFAD mice (Fig. 8e, white bar and Additional file 1: Figure S9b), total cellular IR levels were maintained in 5xFAD//Cre mice (Fig. 8e, grey bar). Furthermore, the assumption that gangliosides may facilitate complex formation between IR and caveolin-1 in Alzheimer’s disease was also corroborated in vivo. 5xFAD mice displayed increased IR/caveolin-1 proximity in cortical neurons, when compared to control mice (Fig. 8f, white bar), whereas IR/Cav-1 proximity was lower in 5xFAD//Cre mice (Fig. 8f, grey bar).

These results suggest that ganglioside reduction as a consequence of GCS inhibition may also protect neuronal IR levels and viability upon Aβ-stress in an Alzheimer’s disease mouse model in vivo.

## Discussion

**Fig. 9:**
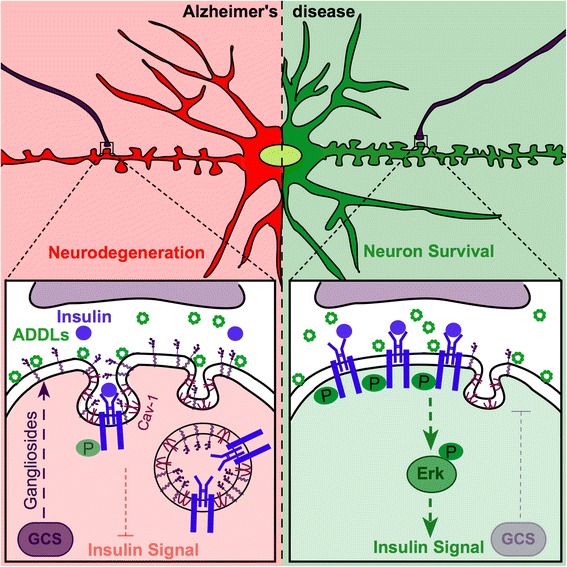
GCS inhibition increases neuronal viability in AD and increases the levels of functional IR on the neuronal cell surface. The proposed mechanism suggests that GCS-derived gangliosides facilitate ADDL-mediated internalization of surface IR via caveolin-1-containing caveolae. Low levels of caveolin-1 in GCS-deficient neurons lead to increased levels of IR at the neuronal surface. IR sensitivity and signaling are maintained in GCS-deficient neurons despite exposure to ADDLs. This contributes to the increased neuronal viability observed in AD models in vivo, where neurons harbor a genetic GCS deletion or in vitro, when they are treated with the specific GCS inhibitor GENZ

Impaired neuronal insulin signaling constitutes a progression factor in the neurodegeneration found in Alzheimer’s disease [11, 15, 29]. Our study now suggests that inhibition of GCS-mediated ganglioside biosynthesis increases the levels of IR at the neuronal surface in primary hippocampal neurons and mHippoE-14 cells. While IR phosphorylation is elevated in ganglioside-depleted ADDL-exposed neurons, specifically the IR-dependent MAPK signaling is increased in ADDL-exposed mHippoE-14 cells. Consequently, GCS inhibition promotes the survival of ADDL-exposed hippocampal cells. Our data furthermore suggests that GCS inhibition also supports survival of cortical neurons and counteracts neurodegeneration in 5xFAD mice in vivo. This work proposes a novel molecular mechanism, suggesting that ADDL-induced loss of surface IR, likely facilitated by caveolin-1, may be prevented upon GCS inhibition. We further suggest that stabilization of IR signaling contributes to increased neuronal resistance towards Aβ stress (Fig. 9), which is in line with studies stating that decreased neuronal IR signaling contributes to neurodegeneration [11, 15, 28, 57].

The precise role of neuronal IR signaling in learning and memory formation is not fully understood [15]. Stimulation of IR signaling is suggested to improve memory in early Alzheimer’s disease [34, 40], even though IR deletion in mouse brain per se does not impair learning and memory, despite increasing Tau phosphorylation [44]. However, it may be assumed that in the case of mice with complete brain IR deletion, compensatory mechanisms involving other insulin signaling–related pathways may prevent memory deficits [15].

Neuronal insulin signaling sustains neuronal survival and plasticity [18]. Oligomeric Aβ species (i.e. ADDLs) mediate rapid internalization of dendritic IR [16], thereby disrupting neuronal IR signaling [57]. Indeed, rodent models of insulin resistance and diabetes display impaired performance in cognitive tests, such as Morris Water Maze [27], which supports an important role of IR signaling for memory and learning. Our work suggests that ADDL-mediated IR loss may be facilitated by the presence of GCS-derived gangliosides. We show that ADDLs induce a dynamic increase in proximity between IR and GD1a in the surrounding membrane microdomains. Furthermore, IR and GD1a co-precipitate with biotinylated ADDLs. We are, however, aware of the fact that co-IP at its best is suitable to detect complex formation of the precipitated substances, which does not necessarily imply direct binding. The concept of complex formation between IR and ADDLs in GD1a-enriched membrane microdomains is moreover supported by triple immune fluorescence.

A potential drawback of long-term pharmacological GCS inhibition is the possibility that side effects may superimpose on the effects that can directly be ascribed to ganglioside loss. Thus, we have investigated a second GCS inhibitor. NB-DNJ exerts a pharmacological mode of GCS inhibition that differs from GENZ. Unlike GENZ, NB-DNJ does not block the binding site for ceramide, but rather mimics the monosaccharide UDP-glucose [49]. Even though NB-DNJ is less effective than GENZ in inhibiting GCS, we observe that NB-DNJ also increases surface IR levels on ADDL-treated neurons. This result suggests that even partial inhibition of GCS may exert positive effects on the surface levels of membrane IR in ADDL-exposed cells. This supports the concept that perturbation of the membrane lipid microenvironment can alter the function of membrane receptors, as also discussed earlier by us [35]. With regard to these results, we conclude that the observed increase in surface IR is a consequence of GCS inhibition.

We have previously demonstrated that GCS-deficient neurons display normal cell viability and normal basal electrophysiological membrane parameters [35]. We observe that GCS inhibition neither leads to less Aβ plaque formation in vivo nor to diminished ADDL binding to neurons in vitro. However, neuronal viability and IR signaling are increased. These findings are consistent with an earlier study showing that the protective effect of insulin treatment on surface IR is mediated by the maintenance of stable IR signaling [16]. We show that pharmacological GCS inhibition stabilizes both surface IR levels and IR signaling in vitro. In line with this, regulatory effects of gangliosides have been suggested for peripheral receptor tyrosine kinases [24], including EGFR [51] and peripheral IR in adipose tissue [1, 25, 52, 55].

We confirm that GENZ treatment protects insulin sensitivity of dendritic IR upon ADDL exposure by PLA. The applicability of PLA to visualize IR tyrosine phosphorylation has been demonstrated earlier both by us [21] and others [12].

Aβ down-regulates MAPK signaling in rat hippocampal neurons [6]. Increases in Grb-2 and ERK1/2 phosphorylation, which enhance synaptogenesis, have been observed in rat hippocampal synaptic membranes after cognitive training [5, 33, 56]. Thus, we suggest that increased IR/ERK1/2 signaling contributes to elevated resistance towards ADDL stress in GENZ-treated neurons.

We show that GCS inhibition leads to a reduction in neuronal caveolin-1 levels. Caveolin-1, which is elevated in AD [20], mediates IR internalization [14, 42, 43]. We demonstrate that loss of surface IR is paralleled by dynamic ADDL-induced complex formation between IR and GD1a as well as caveolin-1 and GD1a. Additionally, caveolin-1 siRNA treatment mimics GENZ-induced IR retention at the neuronal surface. These results are in agreement with a report showing that GD1a increases caveolin-1 expression [50]. Importantly, GCS inhibition does not increase clathrin, which mediates efficient IR signaling [9]. Thus, our results strongly suggest that GCS inhibition may stabilize surface IR as a consequence of decreased caveolin-1 levels and less caveolae formation. Future studies directly investigating IR turnover at ganglioside-deficient membranes can support this hypothesis.

We show that GCS inhibition exerts neuroprotective effects in in vitro and in vivo models of Alzheimer’s disease. Our study allows the tentative conclusion that this positive effect may be attributed to the loss of GD1a. We observe increased ADDL-mediated complex formation between GD1a, IR, and caveolin-1, which coincides with IR loss. Likewise, GT1b shows a similar but less pronounced tendency towards complex formation with IR/caveolin-1 and ADDLs. In fact, GT1b has been ascribed neurotoxic effects in dopaminergic neurons [10], thus making it another interesting target for future studies. GM1 has been suggested as a membranous seed-like structure for monomeric Aβ binding and subsequent aggregation [53]. Since we observe little co-localization of GM1 and dendritically bound oligomeric ADDLs in vitro, we assume that the oligomeric ADDLs generated by us do not depend on binding to GM1. Our results furthermore suggest that GM1 does not influence IR activity and ADDL-mediated internalization. However, further studies are required to elucidate the contribution of individual ganglioside species to ADDL-mediated neurotoxicity.

A part of the neurotoxic effects observed in Alzheimer’s disease has been ascribed to increased activity of sphingomyelinase, which leads to the breakdown of sphingomyelin and a concomitant increase in neurotoxic ceramide [26]. Importantly, neuronal ceramide levels remain unchanged upon GCS deletion, while sphingomyelin levels are elevated [21, 35]. We show that GCS inhibition decreases caveolin-1 levels. Moreover, treatment with GENZ and caveolin-1 siRNA exert similar effects on neuronal surface IR levels. However, sphingomyelin levels are not changed in caveolin-1 siRNA-treated cells. Thus, we surmise that sphingomyelin itself does not cause the observed increase in surface IR. Indeed, gangliosides are suggested to directly influence membrane invagination and endocytotic processes [13]. This rather suggests that neuronal gangliosides down-regulate surface IR levels by maintaining caveolin-1 expression. We furthermore find that the increase in IR/caveolin-1 interactions in our Alzheimer’s disease models in vitro and in vivo is reduced upon GCS inhibition. However, we are aware that decreased IR/caveolin-1 proximity may in part reflect the lower levels of caveolin-1 caused by GCS inhibition. These results suggest an important novel mechanism, which implies that GCS-derived gangliosides are required for ADDL-mediated IR internalization via caveolin-1.

## Conclusion

In conclusion, our study shows that GCS inhibition and subsequent ganglioside reduction enhances neuronal resistance towards Aβ stress in models of Alzheimer’s disease in vitro and in vivo. GCS inhibition increases surface IR levels and thereby ensures efficient IR signal transduction in murine neurons. We show that this increase in surface IR levels is paralleled by decreased caveolin-1 expression in ganglioside-depleted neurons. Furthermore, ADDLs induce dynamic complex formation between the IR and ganglioside GD1a, as well as between caveolin-1 and GD1a. Thus, we hypothesize that lipid microdomains enriched in gangliosides facilitate toxic effects of ADDL on neuronal IR. We therefore propose that ganglioside reduction and subsequent protection of IR signaling contribute to increased resistance towards ADDL stress. Thus, our results also implicate that the reduction of gangliosides may constitute a potential novel target against Alzheimer’s disease.

## Additional file

Additional file 1: Figure S1. Inhibition of ganglioside biosynthesis by GENZ123446 (GENZ) does not affect viability of mHippoE-14 neurons. (a) Immune overlay TLC with antibodies against the indicate ganglioside species confirms that mHippoE-14 cells express the a-series gangliosides GM3, GM1, and GD1a. (b) Morphology of mHippoE-14 cells after GENZ treatment (5 μM GENZ, 7 days), both depicted by phalloidin staining and bright field microscopy. (c) Western blot shows that synaptophysin expression of GENZ-treated mHippoE-14 cells is unchanged (100 nM insulin, 5 min (*n* = 4)). (d) Cell viability of vehicle and GENZ-treated mHippoE-14 cells shows that GENZ treatment itself does not alter cell viability. A positive control (5 % DMSO) verifies the functionality of the MTT assay (Vehicle vs. Genz: *n* = 6; 5 % DMSO *n* = 2-3). **Figure S2.** Generation of neurotoxic amyloid-β_1-42_-derived diffusible ligands (ADDLs). (a) Generation of ADDLs is monitored by electron microscopy. Aβ_1-42_ monomers have been incubated as described in SupplementaryMethods. The subsequent generation of ADDLs and fibrils from the Aβ_1-42_ monomers is shown by electron microscopy. (b) Generation of oligomeric ADDL species is verified by dot blot analysis using the oligomer-specific antibody A11. The 4G8 antibody recognizes all Aβ_1-42_ species. (c) Immunofluorescence depicting that ADDLs (6E10 antibody) bind to mHippoE-14 cells.** Figure S3.** Stimulation with 10nM insulin also increases insulin receptor (IR) tyrosine phosphorylation of GENZ-treated mHippoE-14 cells. (a) Negative control for the IR/phospho-tyrosine (pTyr) proximity ligation assay (PLA; Fig. 2) using only the IR antibody (C-19). (b) A PLA confirms that GENZ treatment enhances insulin-dependent IR tyrosine phosphorylation (IR/pTyr) upon stimulation with insulin (*n* = 37–45 cells). Unpaired two-tailed student’s *t*-test (*p* ≤ 0.001 is marked with (***)); 10nM insulin 3 min. Means ± SEM. Scale bars: 10 μm. **Figure S4.** GCS inhibition increases surface IR levels on mHippoE-14 cells upon ADDL exposure independently of the chemical nature of inhibition. (a) Western blot shows that NMDA receptor levels are not changed by GENZ treatment (*n* = 4). (b) Negative control for PLA stainings (Fig. 3c and e) using one IR antibody only (N-20). (c) Ganglioside expression of mHippoE-14 cells treated with either H_2_O (vehicle) or the GCS inhibitor NB-DNJ (100 μM, 7d, *n* = 4), as shown by TLC. NB-DNJ treatment results in the reduction of individual gangliosides by between approximately 30 to 50 %. (d) A PLA on non-permeabilized mHippoE-14 cells using two different IR antibodies (N-20 and D-17) enables the quantification of IR levels on the neuronal cell surface. Exposure to ADDLs (5 μM, 30 min) leads to a loss of IR in control cells (white bar), whereas surface IR levels are increased on NB-DNJ-treated cells (*n* = 39-101 cells). Unpaired two-tailed student’s *t*-test (if *p* ≤ 0.01 or *p* ≤ 0.001, results are marked with (**) or (***), respectively); 5 μM ADDLs, 30 min. Means ± SEM. Scale bars: 10 μm. **Figure S5.** ADDL treatment has little impact on total IR levels in mHippoE-14 cells. (a) Immunofluorescence of total IR in permeabilized mHippoE-14 cells shows that ADDL exposure has only a slight impact on total IR levels (white bar). The increased total IR levels in GENZ-treated cells reflect the observed effect of GCS inhibition (Fig. 1c) (*n* = 139–145 cells). (b) Negative control of surface IR PLA staining. For the negative control, only one IR antibody (N-20) was used. Unpaired two-tailed student’s *t*-test (if *p* ≤ 0.01 or *p* ≤ 0.001, results are marked with (**) or (***), respectively); 5 μM ADDLs, 24 h. Means ± SEM. Scale bars: 10 μm.** Figure S6.** Increased sphingomyelin expression in GENZ-treated mHippoE-14 neurons is not involved in caveolin-1-mediated up-regulation of surface IR. (a) Thin layer chromatography (TLC) analysis of mHippoE-14 cells shows that GENZ-treated cells display higher sphingomyelin levels (*n* = 4). (b) TLC analysis indicates that mHippoE-14 cells treated with caveolin-1 siRNA, which display increased surface IR levels (Fig. 4c), do not show elevated levels of sphingomyelin (*n* = 4). Unpaired two-tailed student’s *t*-test (*p* ≤ 0.001 was marked with (***)). Means ± SEM. **Figure S7.** Complex formation between IR and ADDLs at dendrites of primary hippocampal neurons involves ganglioside GD1a but not GM1. (a) Immune overlay TLC of a known brain standard confirms the specificity of the antibodies to their respective ganglioside. (b) Both primary hippocampal neurons treated with vehicle and GENZ display immunohistochemically visible synaptic contacts (co-labeling of synaptophysin and phalloidin; arrowheads). Neuronal morphology is furthermore depicted by bright field microscopy. (c) Immune fluorescence indicates that ADDLs (antibody 6E10) partially co-localize with IR on dendrites (phalloidin). (d) Immune fluorescence indicates partial co-localization of ADDLs with phalloidin (white arrowheads). (e) Immunofluorescence shows that dendritic GD1a in part co-localizes with ADDLs, and that GD1a also in part co-localizes with IR. (f) Immunofluorescence shows that dendritic GT1b in part co-localizes with ADDLs, and that GT1b also in part co-localizes with IR. (g) Immunofluorescence shows that dendritic GM1 only co-localizes very little with ADDLs and IR. (h) Negative control for PLA stainings (Fig. 6d) using only one IR antibody (N-20). (i) Combined PLA/phalloidin staining showing the PLA complexes (green labels) on a dendrite of an untreated hippocampal neuron. This staining confirms very little complex formation between IR, ADDLs and ganglioside GM1. (j) Dot blot shows that biotinylated ADDLs co-precipitate with the IR and ganglioside GD1a. (k) Negative control for dendritic caveolin-1/GD1a PLA staining on primary neurons (Fig. 7c and d) using either the caveolin-1 or the GD1a antibody only. 5 μM ADDLs, 30 min. Scale bars = 5 μm. **Figure S8.** Ganglioside reduction by GENZ prevents ADDL-induced IR desensitization. (a) Negative control for dendritic IR/p-Tyr PLA staining on primary neurons (Fig. 7f) using either the IR (C-19) or the p-Tyr antibody only. (b) A PLA using both an IR- and a p-Tyr-specific antibody indicates insulin-evoked dendritic IR phosphorylation (green). ADDL exposure decreases IR phosphorylation (white bar). However, GENZ treatment increases insulin sensitivity of dendritic IR upon ADDL exposure (grey bar). Quantification shows PLA spots/inch dendrite (*n* = 9-13 measurements). Cells were treated with either saline or 10nM insulin for 3 min. Dendrites were visualized with phalloidin. Unpaired two-tailed student’s *t*-test (*p* ≤ 0.001 was marked with (***)). Means ± SEM. Scale bars: 5 μm.** Figure S9.** The 5xFAD (familial Alzheimer’s disease) mouse model with inducible forebrain neuron-specific GCS deletion. (a) Breeding scheme and generation of 5xFAD//Cre mice with forebrain neuron-specific GCS deletion. (b) Negative control of total IR PLA staining on Ugcgf/f mouse brain tissue, using one IR antibody only (N-20). Depicted are cortical neurons. Scale bar: 10 μm. (PDF 13248 kb)
