# Supplementary material for: Lipid microdomain modification sustains neuronal viability in models of Alzheimer’s disease
Source: Acta Neuropathol Commun. 2016 Sep 17;4:103. doi: 10.1186/s40478-016-0354-z (PMC5027102; doi:10.1186/s40478-016-0354-z)
Supplement: Additional file 1: Figure S1. — Inhibition of ganglioside biosynthesis by GENZ123446 (GENZ) does not affect viability of mHippoE-14 neurons. (a) Immune overlay TLC with antibodies against the indicate ganglioside species confirms that mHippoE-14 cells express the a-series gangliosides GM3, GM1, and GD1a. (b) Morphology of mHippoE-14 cells after GENZ treatment (5 μM GENZ, 7 days), both depicted by phalloidin staining and bright field microscopy. (c) Western blot shows that synaptophysin expression of GENZ-treated mHippoE-14 cells is unchanged (100 nM insulin, 5 min (n = 4)). (d) Cell viability of vehicle and GENZ-treated mHippoE-14 cells shows that GENZ treatment itself does not alter cell viability. A positive control (5 % DMSO) verifies the functionality of the MTT assay (Vehicle vs. Genz: n = 6; 5 % DMSO n = 2-3). Figure S2. Generation of neurotoxic amyloid-β1-42-derived diffusible ligands (ADDLs). (a) Generation of ADDLs is monitored by electron microscopy. Aβ1-42 monomers have been incubated as described in SupplementaryMethods. The subsequent generation of ADDLs and fibrils from the Aβ1-42 monomers is shown by electron microscopy. (b) Generation of oligomeric ADDL species is verified by dot blot analysis using the oligomer-specific antibody A11. The 4G8 antibody recognizes all Aβ1-42 species. (c) Immunofluorescence depicting that ADDLs (6E10 antibody) bind to mHippoE-14 cells. Figure S3. Stimulation with 10nM insulin also increases insulin receptor (IR) tyrosine phosphorylation of GENZ-treated mHippoE-14 cells. (a) Negative control for the IR/phospho-tyrosine (pTyr) proximity ligation assay (PLA; Fig. 2) using only the IR antibody (C-19). (b) A PLA confirms that GENZ treatment enhances insulin-dependent IR tyrosine phosphorylation (IR/pTyr) upon stimulation with insulin (n = 37–45 cells). Unpaired two-tailed student’s t-test (p ≤ 0.001 is marked with (***)); 10nM insulin 3 min. Means ± SEM. Scale bars: 10 μm. Figure S4. GCS inhibition increases surface IR levels on mHippoE-14 cells upon ADDL [file 40478_2016_354_MOESM1_ESM.pdf]

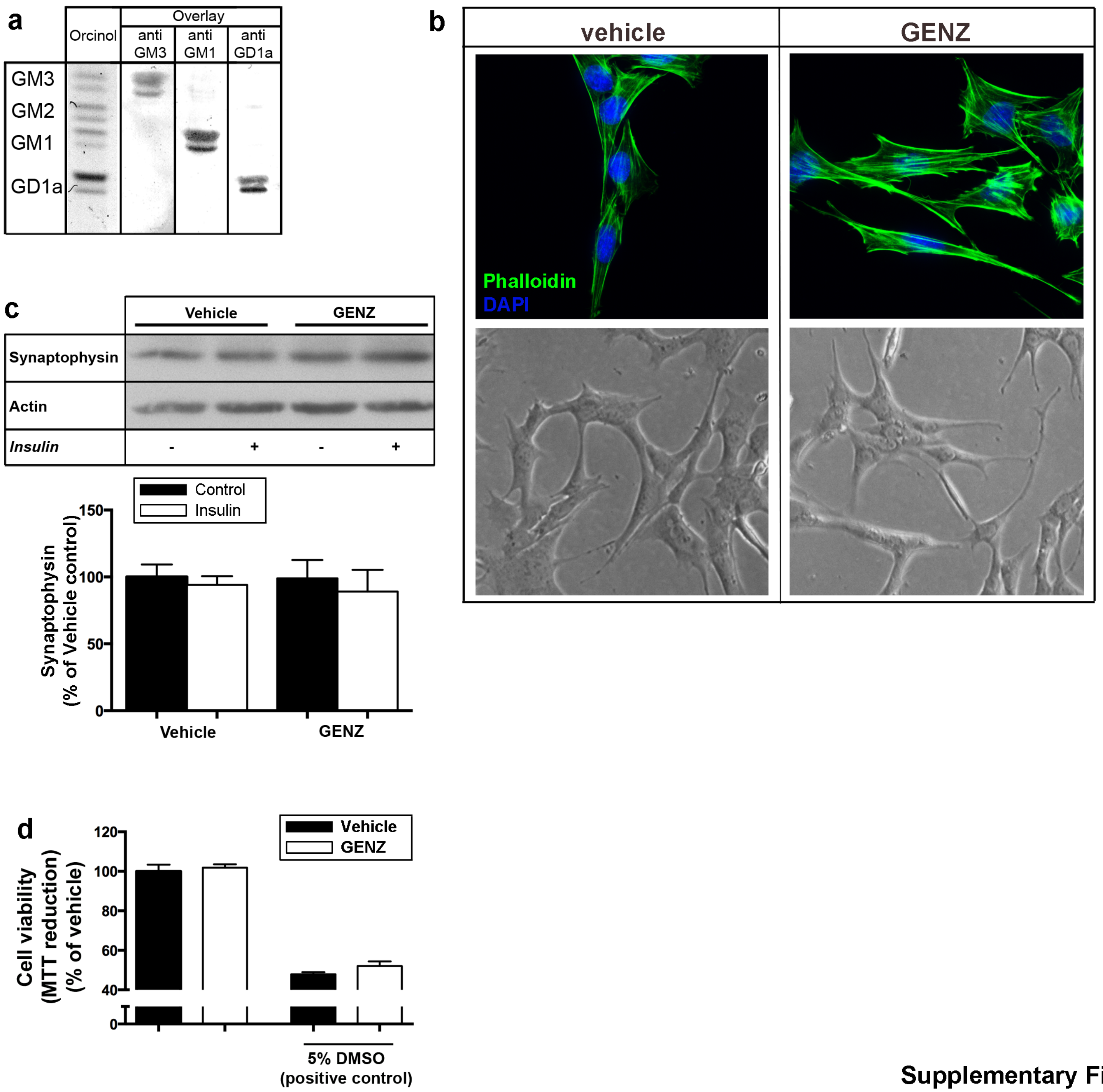

Supplementary Fig. 1

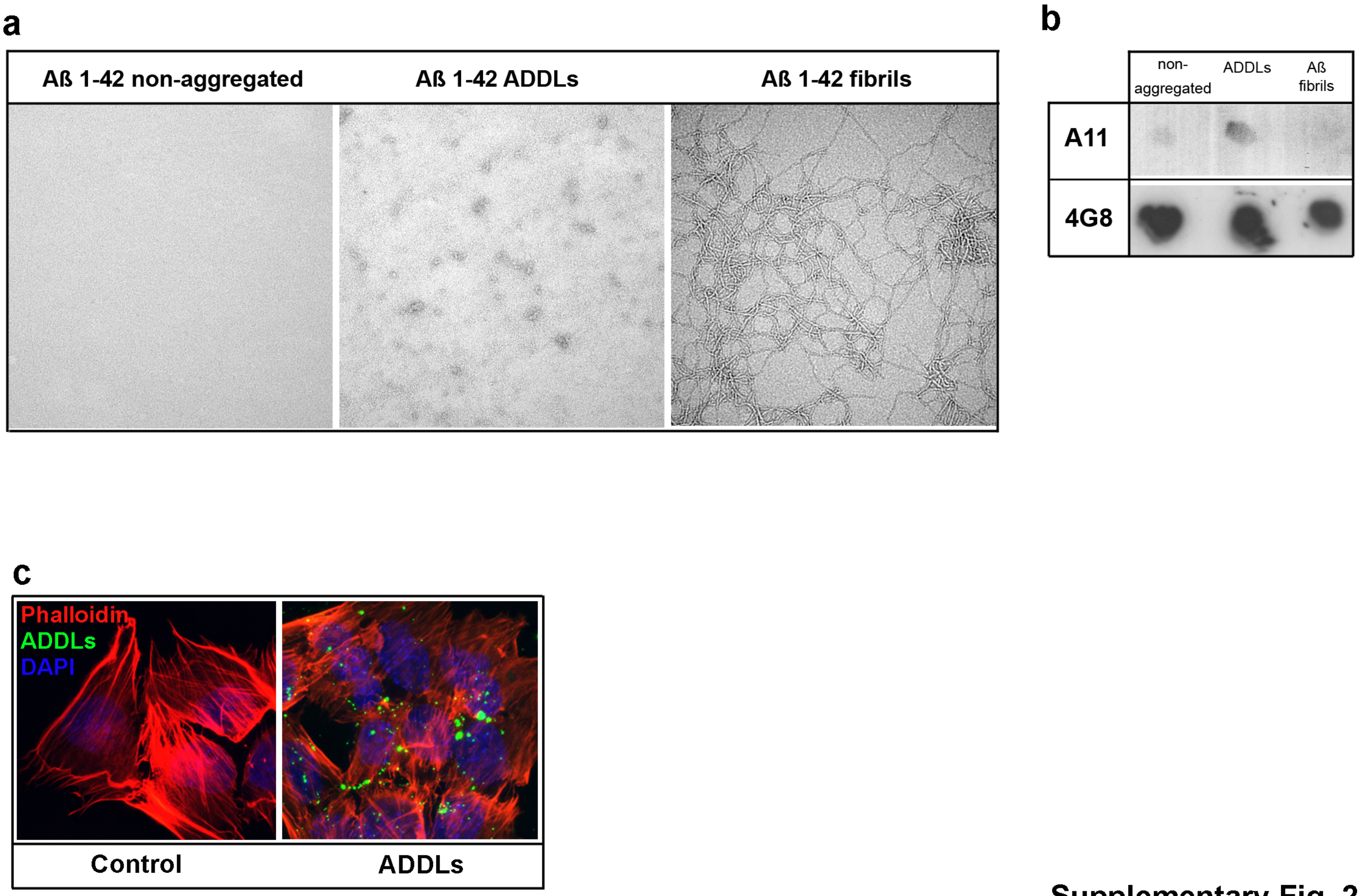

Supplementary Fig. 2

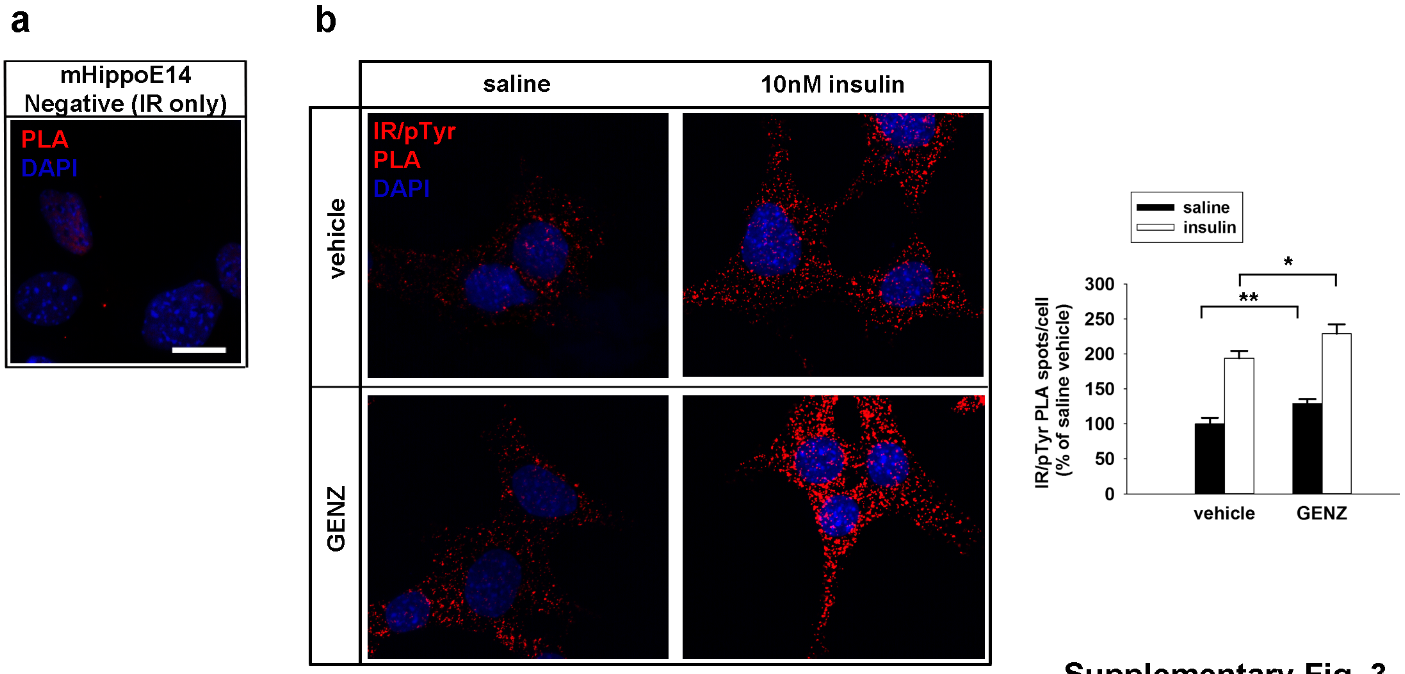

Supplementary Fig. 3

Supplementary Fig. 4

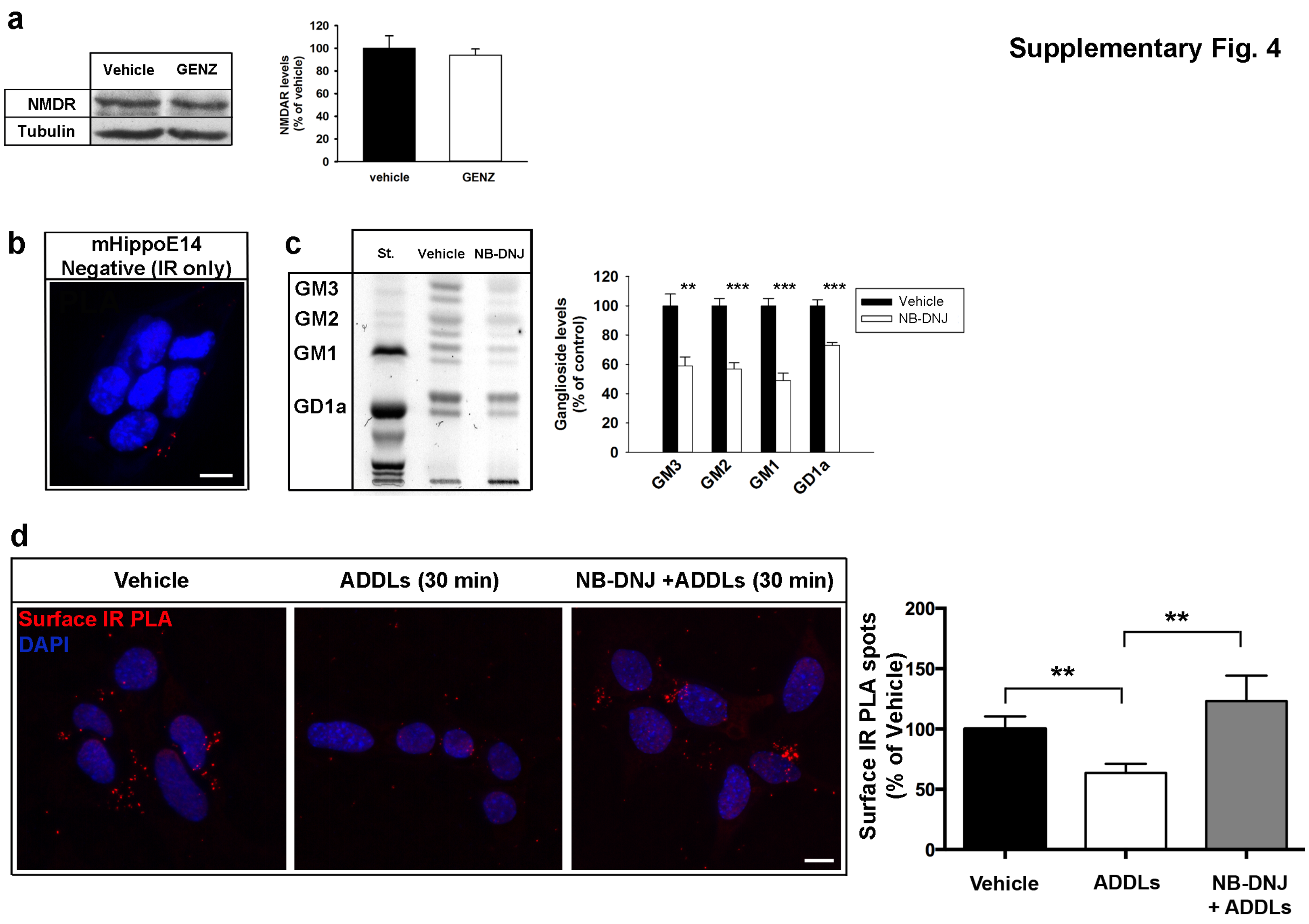

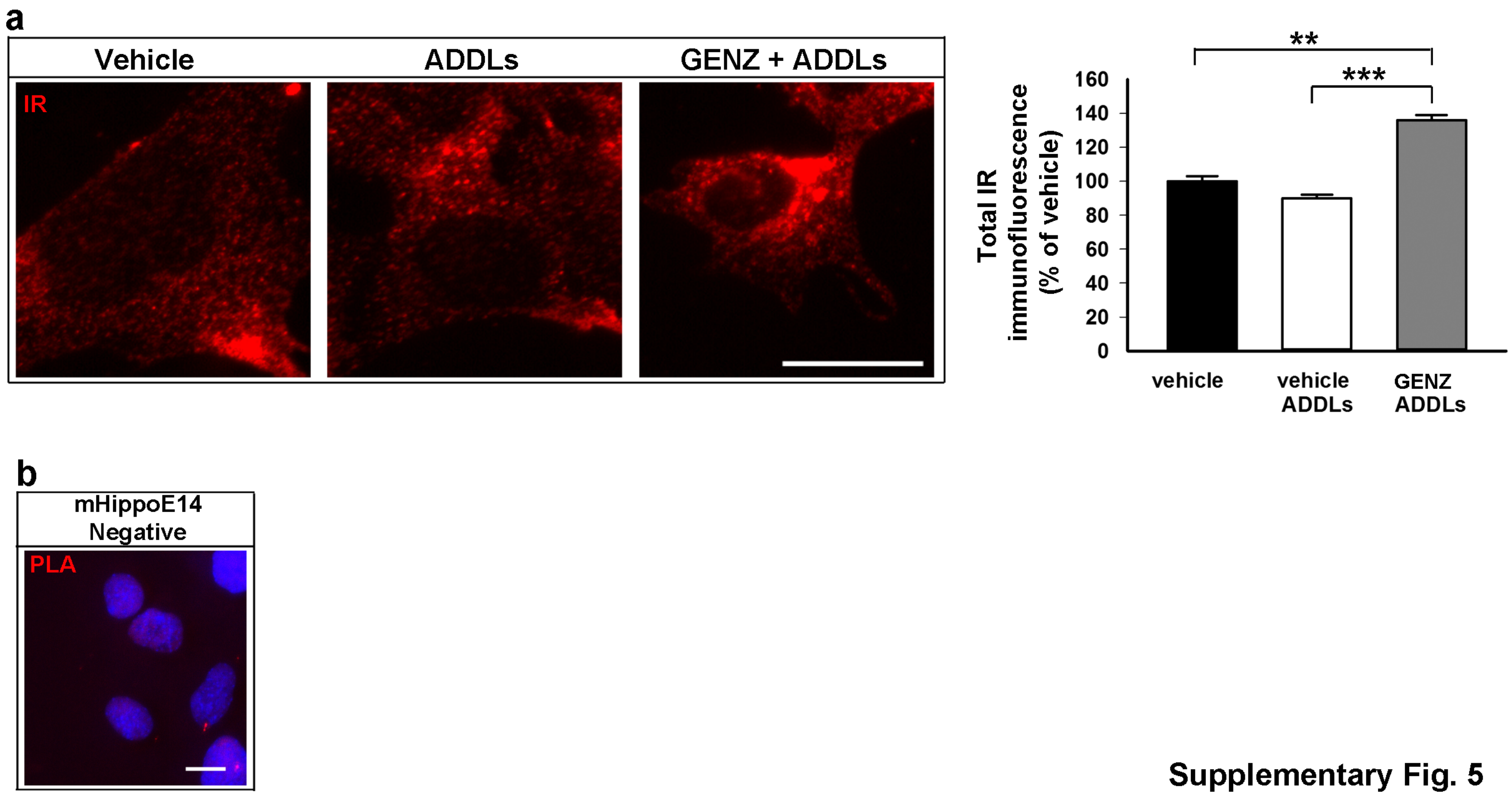

Supplementary Fig. 5

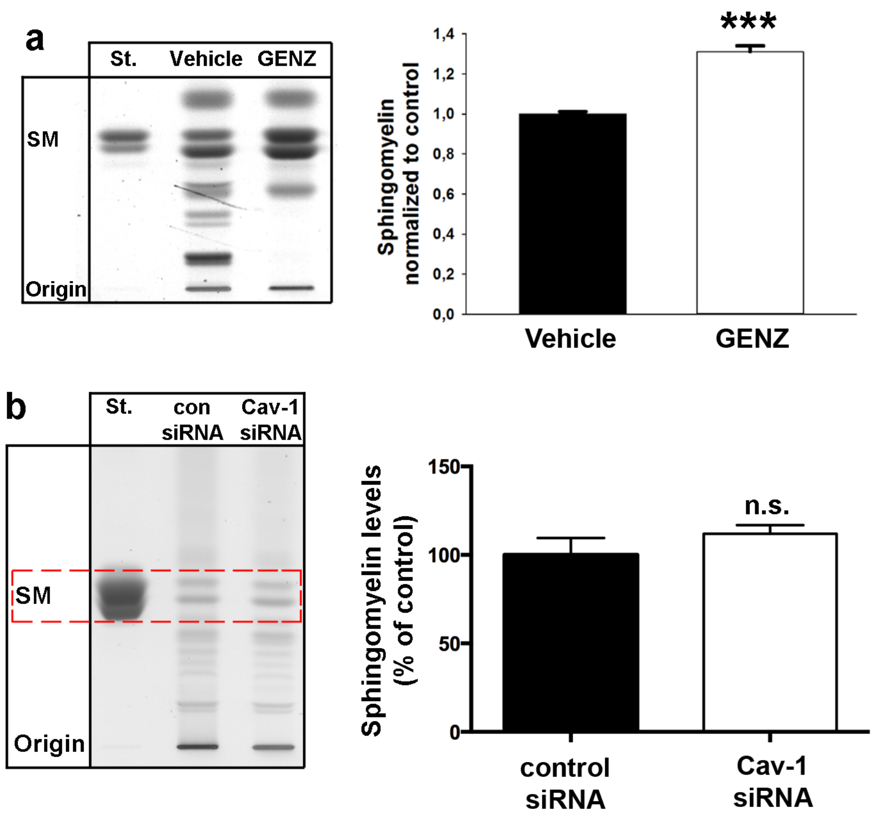

Supplementary Fig. 6

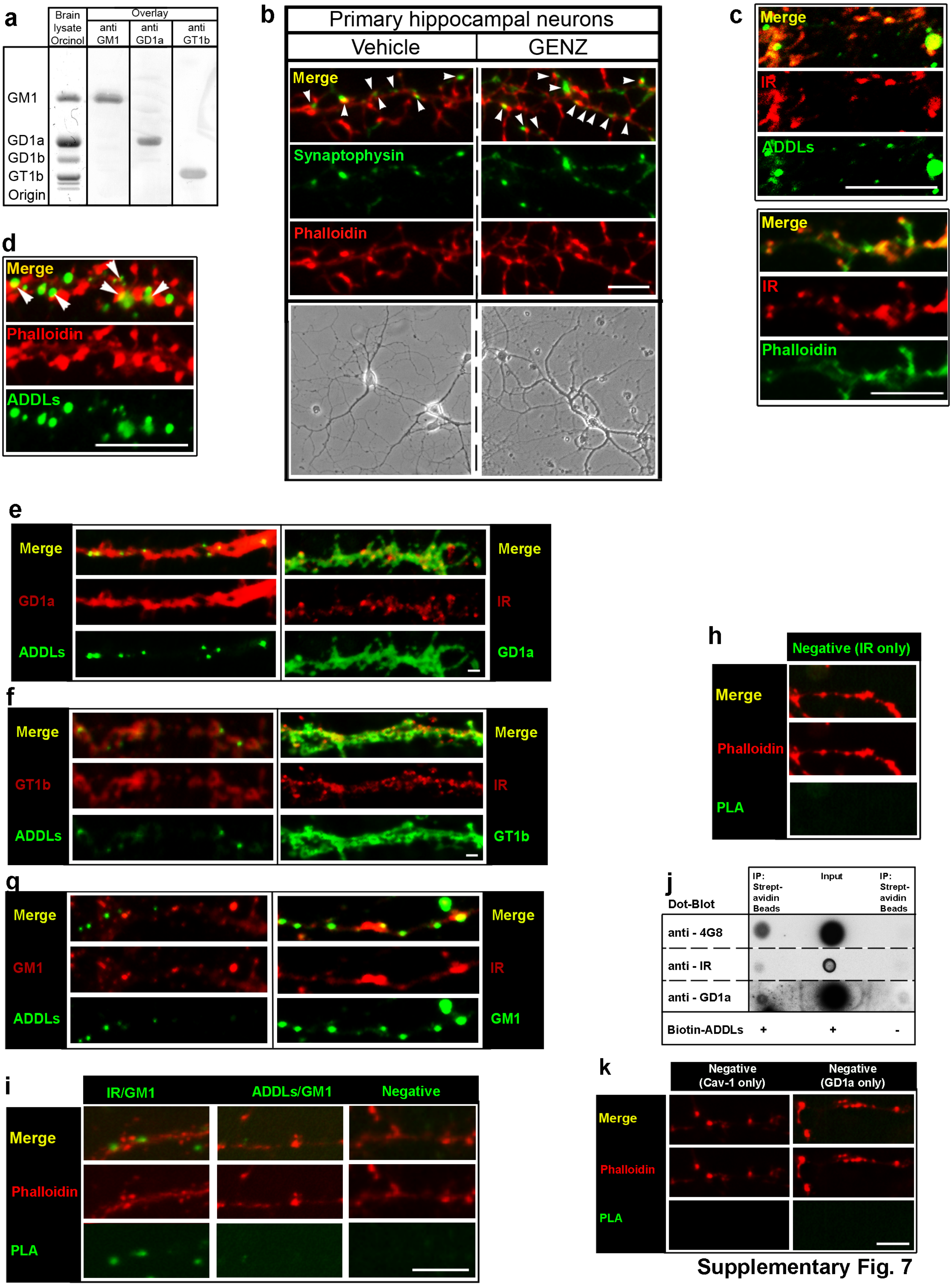

Supplementary Fig. 7

Supplementary Fig. 8

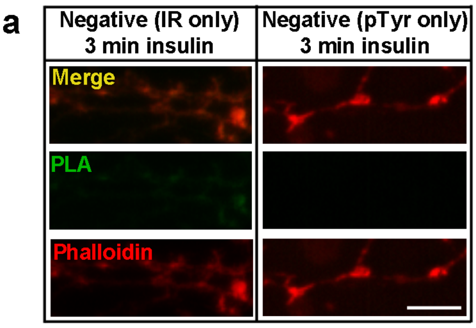

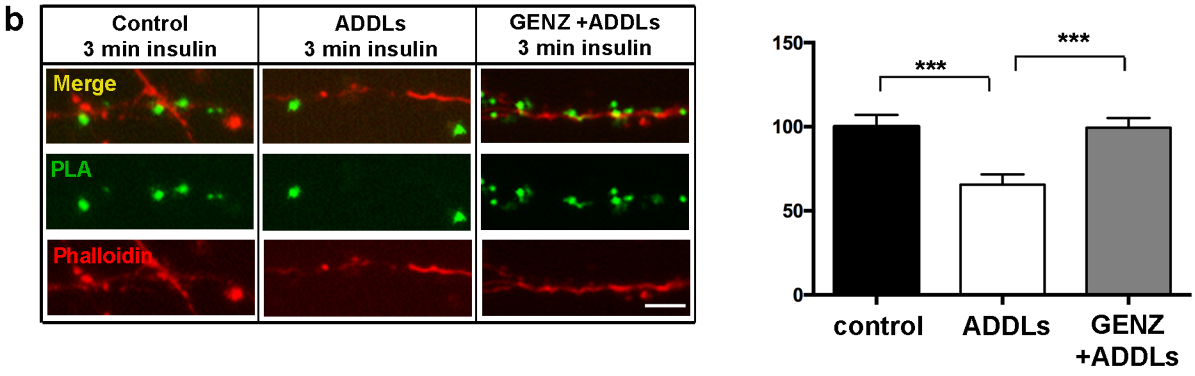

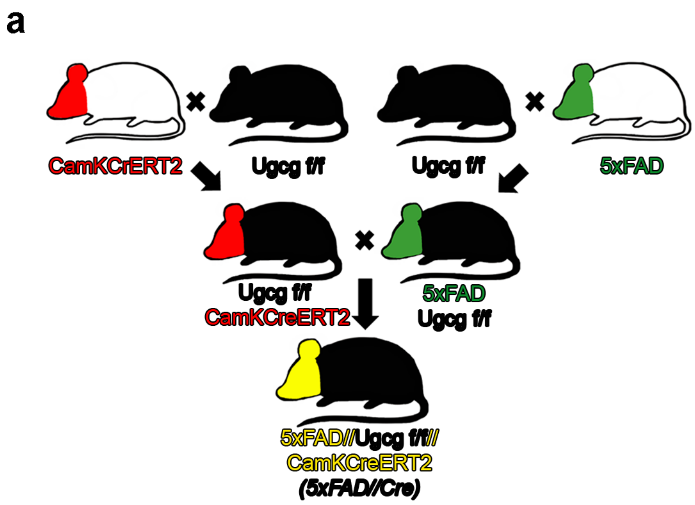

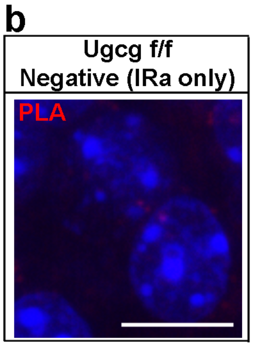

Supplementary Fig. 9
